# Lifetime analysis with monotonic degradation: a boosted first hitting time model based on a homogeneous gamma process

**DOI:** 10.1007/s10985-025-09648-z

**Published:** 2025-04-05

**Authors:** Clara Bertinelli Salucci, Azzeddine Bakdi, Ingrid Kristine Glad, Bo Henry Lindqvist, Erik Vanem, Riccardo De Bin

**Affiliations:** 1 Department of Mathematics, University of Oslo, Moltke Moes vei 35, 0851 Oslo, Norway; 2 Corvus Energy, Tormod Gjestlands veg 51, 3936 Porsgrunn, Norway; 3 Department of Mathematical Sciences, Norwegian University of Science and Technology, Alfred Getz vei 1, 7491 Trondheim, Norway; 4 DNV Group Technology and Research, Veritasveien 1, 1322 Høvik, Norway

**Keywords:** First hitting time, Gradient boosting, Homogeneous gamma process, Cox model, Time-to-event outcome

## Abstract

In the context of time-to-event analysis, First hitting time methods consider the event occurrence as the ending point of some evolving process. The characteristics of the process are of great relevance for the analysis, which makes this class of models interesting and particularly suitable for applications where something about the degradation path is known. In cases where the degradation can only worsen, a monotonic process is the most suitable choice. This paper proposes a boosting algorithm for first hitting time models based on an underlying homogeneous gamma process to account for the monotonicity of the degradation trend. The predictive power and versatility of the algorithm are shown with real data examples from both engineering and biomedical applications, as well as with simulated examples.

**Supplementary Information:**

The online version contains supplementary material available at 10.1007/s10985-025-09648-z.

## Introduction

A common trait of the variety of statistical methods developed for time-to-event data analysis, among which the Cox model is indisputably the most studied and applied, is that they focus merely on the event itself: they do not take into account that the event comes as ending point of some evolving process, often unknown and hence neglected (Aalen and Gjessing 2001). Conversely, first hitting time (FHT) models are built on the principle that the event occurs when an underlying stochastic process reaches a threshold, or boundary, for the first time (Caroni 2017). The underlying process is thus in the spotlight: choosing a suitable process becomes crucial for the analysis, as the assumptions made on the process will determine the event-time distribution. Indeed, the need for a specific distributional assumption makes the approach less flexible than, for example, the Cox model. On the other hand, proportional hazards are not needed for FHT models to hold.

Many fields have seen the successful application of FHT models with promising results, from the biomedical domain to econometrics, finance, physics and geophysics, and engineering. In particular, Caroni (2017) affirms that biostatistics and engineering are the main application fields: in the first case, the usual scenario is the study of patients’ survival (Aalen and Gjessing 2005; Lee et al. 2004, 2008); in the second, survival of machines or components, often called *reliability* or *remaining useful life* (RUL) estimation (Park and Padgett 2006; Hasilová and Vališ 2018; Wang et al. 2019). In reliability analysis, degradation models commonly involve gamma processes, due to their monotonicity: many examples can be found in van Noortwijk (2009) and Si et al. (2011). In the context of biostatistics, Wiener processes provide powerful tools for time-to-event data analysis. Since the Wiener process is characterised by Gaussian increments, these processes are not guaranteed to be monotonic, which on the other hand may make them less suitable for cases concerning irreversible degradation (e.g. nontreatable cancer, fatigue accumulation, growth of a crack).

De Bin and Stikbakke (2022) have proposed a boosting algorithm for FHT models based on a Wiener process with drift. The aim of this work is to follow their direction and propose an extension of the boosting algorithm for first hitting time process based on an underlying gamma process, suitable for both biomedical and engineering purposes. To our knowledge, this is the first time that a boosting algorithm is developed to fit a gamma-based FHT model: in fact, choosing a gamma process entails dealing with a rather complicated FHT distribution, its survival function and derivatives, which is not an easy task. The aim of this work is providing a valid alternative to other lifetime methods for any kind of application characterised by a monotonic trend, be it low- or high-dimensional.

The remainder of this article is organised as follows: Sect. 2 begins with an overview of FHT models and gradient boosting and provides our algorithm for a boosted FHT model based on an underlying homogeneous gamma process; Sect. 3 presents the results we have obtained in simulated and real-data examples from both engineering and biomedical applications; Sect. 4 concludes the paper with final remarks.

## Methods

### Overview

#### First hitting time models

First hitting time models are methods for time-to-event analysis in which the event of interest is considered to happen at the first passage time *T* of an underlying stochastic process to a threshold or, more generically, a boundary set $\mathcal{B}$:

1 $$\begin{aligned} T = \inf \{t: Y(t) \in \mathcal {B} \}. \end{aligned}$$

The underlying process *Y*(*t*) describes the evolution of the process that ultimately leads to the event itself, from a given initial value $Y(0) = y_0 \not \in \mathcal {B}$ to the ending point. In the context of this paper, *Y*(*t*) is taken to be a *health process*, i.e. it represents the health of some entity—be it a human being or an inanimate object such as an electric battery - that deteriorates over time until a critical point is reached, determining the *End of Life* (EoL) occurrence. In particular, in this work we consider examples from both biomedical and engineering applications, hence the process will pertain to both individuals affected by cancer and lithium-ion batteries cycled in laboratory until exhaustion. Generally speaking, the health process can be either observable or unobservable. For the applications we consider, it is reasonable to deem it unobservable: in fact, in the case of oncologic patients we clearly do not have any specific “health measurement”, but we only observe the time-to-event along with clinic or genetic covariate measurements; in the case of electric batteries we could in principle have observed the so-called *State of Health* (SoH), but obtaining such a measurement requires specific tests that are expensive and disruptive: as a result, the most common situation in this application field is observing time-series of sensor data from the batteries, which will be used to extract covariates for the models, without any (or with very seldom) SoH measurements.

The choice of the process is evidently of great importance, as it determines the form of the FHT model. Even when *Y*(*t*) is unobservable, assumptions on the characteristics of the degradation can, and have to, be made. For example, De Bin and Stikbakke (2022) choose an underlying Wiener process as it is assumed that, despite having a decreasing trend over time, the health of an individual fluctuates up and down. Additionally, the Wiener process is relatively easy to work with from a mathematical point of view: it is the most widely employed model for this reason, too. In many cases, however, the phenomena driving the degradation are irreversible, therefore a monotonic process is the most suitable alternative.

#### Gradient boosting

Boosting is an ensemble method in which weak learners are sequentially combined to form a strong learner. The algorithm, which is rather general and can be implemented in a variety of ways, was originally developed as a “black-box” machine learning method (Schapire 1990; Freund 1995), and later interpreted statistically as a forward stagewise additive modelling method (Friedman et al. 2000). The common characteristic to any kind of boosting algorithm is that the model is built sequentially by base learners that, at each iteration, aim at improving the estimate by compensating for the flaws of the previous ones, e.g. by giving more weight to misspecified observations in a classification task. The advancement is carried out in small steps, i.e. using “weak” learners. Specifically in gradient boosting, the strategy is to train the model on the generalised residuals of the previous step, i.e. the negative gradient of the loss function. The importance of the weakness of the base learners has been discussed and proven in Bühlmann and Yu (2003) and Hastie et al. (2009): common choices are ordinary linear regression models, splines, or stumps (one-level trees), suitably “penalised”. The predictive power of gradient boosting comes from its capability of reaching an optimal bias-variance trade-off: this is achieved by stopping the algorithm at the best number of iterations $m_{\tiny {\hbox {stop}}}$, which represents the main tuning parameter of boosting together with the step-size, or shrinkage parameter, $\nu$ (Friedman 2001). The default choice is to set $\nu = 0.1$ and find the optimal $m_{\tiny {\hbox {stop}}}$ through cross-validation (Friedman 2001; Bühlmann and Hothorn 2007), as the two parameters are obviously related (Seibold et al. 2018).

Adopting a boosting method is particularly convenient in high-dimensional problems (when the number of variables is larger than the number of observations), which often recur in biomedical applications where omics variables are involved. In such cases, it is reasonable to use a *componentwise* version of boosting, which explores only one dimension of the sample space at each step, the one corresponding to the best improvement for the current iteration. As a consequence, a natural variable selection mechanism takes place. In fact, as long as the stopping point $m_{\tiny {\hbox {stop}}}$ is adequately selected, the variables that are not significant for predicting the outcome will never enter the model. The generic algorithm for componentwise boosting with a parametric learner of the form $h = f(X; \zeta )$ is provided in Algorithm 1; the version adopted to boost our FHT model based on an underlying gamma process will be specified in Sect. 2.2.2. Once a loss function $L(y, f(X, \zeta ))$ is specified, initial values are assigned to the parameter vector. Then, at each iteration *m* the negative gradient *u* of the loss function is computed and evaluated at the previous step solution,

2 $$\begin{aligned} u = - \frac{\partial L(y, f(X, \zeta ))}{\partial f(X,\zeta )} \biggr \vert _{\zeta =\hat{\zeta }^{[m-1]}}, \end{aligned}$$

where *y* is the *n*-dimensional response vector, *X* is the $n \times (p+1)$ matrix of covariates, (*y*, *X*) constitute the training set, and $\zeta = (\zeta _0,\ldots , \zeta _p)$ is the $(p+1)$-dimensional parameter vector. In the following, the notation $\zeta _j$ and $x_j$ will refer to the *j*th element of the vector $\zeta$ and the *j*th row of the matrix *X*, respectively.

The base learner is then fit to the negative gradient vector for all the components of the parameter vector. We denote the resulting *n*-dimensional vector as $\hat{h}_j(u,x_j)$, where the index $j \in \{0, 1,\ldots , p\}$ indicates that the base learner $\hat{h}(\cdot )$ is fitted on the *j*th dimension; then, the best update (e.g. the one that minimises the loss function), here denoted by $j^\star$, is selected and the estimate is updated as

3 $$\begin{aligned} \hat{\zeta }_j^{[m]}&= {\left\{ \begin{array}{ll} \hat{\zeta }_j^{[m]} + \nu \hat{h}_{j^\star }(u, x_j^\star ) & \text {if } j = j^\star , \\ \hat{\zeta }_j^{[m - 1]} & \text {if } j \ne j^\star \end{array}\right. } \end{aligned}$$

for any iteration $m = 1,\ldots , m_{\tiny {\hbox {stop}}}$.

The shrinkage parameter $\nu$ controls the amount of shrinkage, i.e. it penalises the base learner, and prevents overfitting. At the last iteration $m_{\tiny {\hbox {stop}}}$, chosen via cross-validation, the final estimate for the parameter vector is $(\hat{\zeta }_0^{[m_{\tiny {\hbox {stop}}}]},\ldots , \hat{\zeta }_p^{[m_{\tiny {\hbox {stop}}}]})$ with

4 $$\begin{aligned} \hat{\zeta }_{j}^{[m_{\tiny {\hbox {stop}}}]} = \hat{\zeta }_{j}^{[m_{\tiny {\hbox {stop}}}-1]} + \nu \hat{h}_{j}(u, x_{j}) \cdot \mathbbm {1} (j = j^\star ) \end{aligned}$$

for $$j=0,\ldots ,p$$.

**Algorithm 1:**
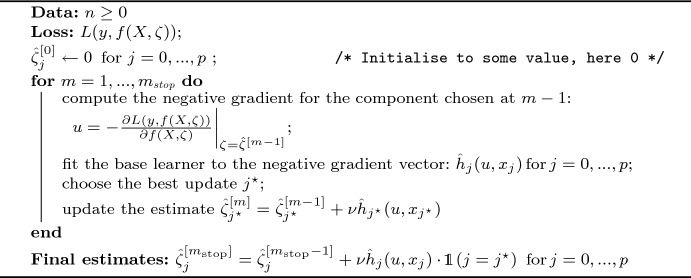
Componentwise gradient boosting with parametric learner

### Boosted first hitting time models based on a gamma process

#### The gamma process as underlying process

We assume that the deterioration leading to the event occurrence is well described by a homogeneous gamma process $\{D(t), t> 0\}$ with shape function $\alpha (t) = at$, for constant $a> 0$, and constant scale parameter $\beta> 0$. The process *D*(*t*) is characterised by independent increments $D(t) - D(s)$ that are gamma distributed with shape $\alpha (t - s)$ and scale $\beta$, for every $0 \le s < t$; moreover, $D(0) = 0$ with probability 1. The increments are also stationary, since *D*(*t*) and $D(t+\delta ) - D(\delta )$ have the same distribution for any *t* and $\delta$. Assuming this deterioration process with a constant threshold $c> 0$, the FHT cumulative distribution function (cdf) for the first hitting time variable $T_c = \hbox {inf}\{t \ge 0: D(t) \ge c\}$ can be directly recovered (Park and Padgett 2005; Paroissin and Salami 2014) as

5 $$\begin{aligned} F_{T_c}(t; a, \beta ) = P(T_c \le t) = 1 - P(T_c> t) = 1 - P(D(t) < c) = \frac{\varGamma (at, c\beta )}{\varGamma (at)}, \end{aligned}$$

where $\varGamma (p,q) = \int _{q}^{\infty } z^{p-1} \, e^{-z} \, dz$ is the upper incomplete gamma function, and $\varGamma (p) = \int _{0}^{\infty } z^{p-1} \, e^{-z} \, dz$ is the (complete) gamma function.

In order to have an intuitive interpretation of the parameters of the model, note first that *D*(*t*) for fixed *t* is gamma distributed with density

6 $$\begin{aligned} f_{D(t)}(x; a, \beta ) = \frac{\beta }{\varGamma (at)}(\beta x)^{at-1}e^{-\beta x}; \, \, x> 0. \end{aligned}$$

It follows that

7 $$\begin{aligned} E[D(t)] = \frac{at}{\beta }, \end{aligned}$$

so that $a/\beta$ is the “expected” slope of the process *D*(*t*). It appears to be harder to find $E[T_c]$—see e.g. Kahle et al. (2016), Section 2.6.2 for an exact expression. An approximation reported by Paroissin and Salami (2014) is

8 $$\begin{aligned} E[T_c] \approx \frac{c\beta + \frac{1}{2}}{a}. \end{aligned}$$

It results from Eq. (5) that the distribution of $T_c$ depends on $\beta$ and *c* only through their product $\beta c$. Hence, the scale parameter $\beta$ is identifiable only when *c* is known (fixed). Park and Padgett (2005) resolve this apparent dilemma by introducing a parameter $c_\beta = c\beta$. In the following, we will instead fix $c=1$ and regard $\beta$ as the parameter of interest. We then obtain the pdf by differentiating equation (5) with respect to *t*,

9 $$\begin{aligned} f_{T}(t; a, \beta ) = \frac{a \biggl [ G^{3,0}_{2,3} \left( \beta \, \bigg | \begin{matrix} 1, 1 \\ 0, 0, a t \end{matrix} \right) + \varGamma (at, \beta )(\ln (\beta ) - \psi ^{(0)}(at)) \biggr ]}{\varGamma (at)} \end{aligned}$$

where $\psi ^{(0)}(\alpha )$ is the digamma function, and the *Meijer G-function* is a complex-valued integral (Meijer 1936) defined by

10 $$\begin{aligned} G^{m,n}_{p,q}\biggl (z \bigg \vert \begin{matrix} r_1,\ldots , r_p \\ v_1,\ldots , v_q \end{matrix} \biggr ) = \frac{1}{2\pi i} \int _L \frac{\prod _{j=1}^{m} \varGamma (v_j-s) \prod _{j=1}{n} \varGamma (1-r_j + s)}{\prod _{j=m+1}^{q} \varGamma (1-v_j+s) \prod _{j=n+1}{p} \varGamma (r_j - s)} z_s \, ds. \end{aligned}$$

We provide a proof of Eq. (9) in “Appendix 1.1”. Alternatively, the pdf can be expressed in terms of a generalised hypergeometric series of order (2,2) as in Park and Padgett (2005), Paroissin and Salami (2014), Sildnes and Lindqvist (2018),

11 $$\begin{aligned} f_{T}(t; a, \beta )&=\ a \left( \psi ^{(0)}(at) - \ln (\beta ) \right) \frac{\gamma (at, \beta )}{\varGamma (at)} \end{aligned}$$

12 $$\begin{aligned}&\quad + \frac{a}{a^2 t^2 \varGamma (at)} (\beta )^{at} \, _2F_2\left( at, at; at+1, at+1; -\beta \right) \end{aligned}$$

where $\gamma (p,q) = \int _{0}^{q} z^{p-1} \, e^{-z} \, dz$ is the lower incomplete gamma function, and

13 $$\begin{aligned} _pF_q(r_1,\ldots ,r_p; v_1,\ldots ,v_q; z) = \sum _{k=0}^\infty \frac{(r_1)_k,\ldots ,(r_p)_k}{(v_1)_k,\ldots ,(v_q)_k} \cdot \frac{z^k}{k!} \end{aligned}$$

with $$(a_i)_k = a_i \cdot (a_i + 1) \cdot \cdot \cdot (a_i + k - 1)$$.

The survival function *S*(*t*) can be recovered from the cdf,

14 $$\begin{aligned} S_T(t; a, \beta ) = 1 - F_T(t; a, \beta ) = 1 - \frac{\varGamma (at, \beta )}{\varGamma (at)}. \end{aligned}$$

We remark that the FHT cdf in Eq. (5), though similar to the inverse gamma cdf $\frac{\varGamma (\alpha (t), \frac{\beta }{t})}{\varGamma (\alpha (t))}$, is *not* inverse gamma. The FHT pdf in both forms (9) and (12) is cumbersome, and the corresponding gradients are even more so. Nevertheless, the pdf is essential for constructing the log-likelihood, and the log-likelihood gradients are needed for the boosting algorithm. Therefore, in constructing our FHT boosting model, it is advisable to find a suitable approximation, which we provide in Sect. 2.2.2.

##### Censoring

In time-to-event data analysis one often has to take into account that some of the observations may be *right-censored*, i.e. some of the individuals do not experience the event throughout the study and the observed time *t* is just the last follow-up time rather than the time-to-event. In this case, the log-likelihood function must take into account the presence of censored, or *incomplete*, observations. Let us consider $d_i \in \{0,1\}$ for $i=1,\ldots ,n$ as a censoring indicator, which is 0 for censored observations and 1 for uncensored, or complete, observations. The log-likelihood for *n* individuals (let from now on the term *individual* denote either a person or an object under study) in presence of censoring is then

15 $$\begin{aligned} \ell (a, \beta ) = \sum _{i=1}^n \biggl \{ d_i \, \bigl [ \ln f(t_i; a, \beta ) \bigr ] \, + (1-d_i) \bigl [ \ln S(t_i; a, \beta ) \bigr ] \biggr \}. \end{aligned}$$

##### Regression

In the presence of a $n \times (p+1)$-dimensional covariate matrix *X* (e.g., for the applications considered here, a matrix of measurements of battery parameters or clinical and molecular measurements for individuals), the first column of which is a vector of elements all equal to 1, we can specify a dependency of the model parameters on *X*, as is common practice to do. Since both the parameters are strictly positive, we use a logarithmic link for both:

16 $$\begin{aligned} \begin{aligned} a(X; \theta ) = \exp (X\theta ) \, \,&\implies \, \, \ln a(X; \theta ) = X\theta \\ \beta (X; \gamma ) = \exp (X\gamma ) \, \,&\implies \, \, \ln \beta (X; \gamma ) = X\gamma , \end{aligned} \end{aligned}$$

where $\theta$ and $\gamma$ are $(p+1)$-dimensional vectors of the form $\theta = (\theta _0,\ldots , \theta _p)$ and $\gamma = (\gamma _0,\ldots , \gamma _p)$, and exp$(X\theta )$, exp$(X\gamma )$ refer to componentwise exponentialisation. The logarithmic link function is widely used because it guarantees positivity while remaining simple, smooth, and compatible with gradient-based optimisation methods. However, alternatives like the softplus function $\ln (1+e^x)$ or reparametrisation methods could also be considered.

Regressing the parameters of the stochastic process on some covariate matrix is a widely used and standard approach to introduce heterogeneity among individuals. While we consider this strategy acceptable for this stage of our analysis, there are instances where allowing for a greater degree of heterogeneity may be necessary: exploring this possibility is an interesting direction for future development of this method. For example, in certain cases, it may not be reasonable to assume that all individuals share the same threshold or starting level. In such situations, considering a random threshold as in Paroissin and Salami (2009) could be beneficial. Another potential development is to include an individual random effect in the regression equation, following the approach of Wang et al. (2021).

#### Boosting the model

The boosting algorithm is implemented in R (Core Team 2021), using as in De Bin and Stikbakke (2022) the R package gamboostLSS (Hofner et al. 2016) that was specifically developed for generalised additive models for location, scale and shape (Rigby and Stasinopoulos 2005). The package offers two different strategies for the model update: in the case of a two-parameter model, here *a* and $\beta$, the *cyclic* version updates at each iteration one parameter using the current fit of the other as offset, rotating between the two; while the *noncyclic* version selects at each iteration the update of a base-learner for the parameter that best fits the negative gradient. Both versions of the algorithm were evaluated in the real data examples, and their results are compared in Sect. 3.

Algorithm 2 is our algorithm for boosting the FHT model. As loss function, we employ the FHT negative log-likelihood as specified in Eq. (15) with parameters depending on covariates as specified in Eq. (16),

17 $$\begin{aligned} L(y, f(X; \theta , \gamma )) \, = \, - \ell \bigl (\exp (X\theta ), \exp (X\gamma )\bigr ). \end{aligned}$$

We choose either linear effects or stumps as base learners, and initialise the parameters to the maximum likelihood estimates for *a* and $\beta$,

18 $$\begin{aligned} (\hat{\theta }_{0}, \hat{\gamma }_{0}) = {{\,\textrm{argmax}\,}}_{a, \beta } \ell (a, \beta ). \end{aligned}$$

Note that the choice of initial values for the parameters in the maximum likelihood maximisation can have a rather significant effect on the success of the algorithm. Additionally, the two parameters can have different optimal numbers of iterations, $m_{\tiny {\hbox {stop}}}^{\alpha }$ and $m_{\tiny {\hbox {stop}}}^{\beta }$, which are selected through five-fold cross-validation.

As briefly aforementioned, implementing the FHT pdf and its gradients is not easy, mainly due to the presence of the incomplete gamma function and its derivatives. In fact, when it comes to the negative gradients in Eq. (2), their exact expressions are (proofs in “Appendices 1.2 and 1.3”):

19 $$\begin{aligned} u_a = \frac{ \left( \begin{aligned}&-\left( at \psi ^{(0)}(at) + 1\right) \left( G_3 + \varGamma (at, \beta )\left( \ln \beta - \psi ^{(0)}(at)\right) \right) \\ &\quad + at\left( 2G_4 + \ln \beta G_3\right) \\ &\quad + at\left( \ln \beta - \psi ^{(0)}(at)\right) \left( G_3 + \ln \beta \varGamma (at, \beta )\right) \\&\quad - at \psi ^{(1)}(at)\varGamma (at, \beta ) \end{aligned} \right) }{ a\left( G_3 + \varGamma (at, \beta )\left( \ln \beta - \psi ^{(0)}(at) \right) \right) } \end{aligned}$$

and

20 $$\begin{aligned} u_\beta = \frac{-e^{-\beta } \beta ^{at-1} (\ln \beta - \psi ^{(0)}(at)) + \frac{\varGamma (at, \beta )}{\beta } - \frac{\pi \bigl ( \frac{\csc (\pi at)}{\varGamma (1-at)} - \frac{at \csc {\pi at} \varGamma (at, 0, \beta )}{\varGamma (1-at)\varGamma (1+at)} \bigr )}{\beta }}{G_3 + \varGamma (at, \beta )(\ln \beta - \psi ^{(0)}(at))}, \end{aligned}$$

where, for the sake of readability, we have set $$G_3 = G^{3,0}_{2,3} \left( \beta \, \bigg | \begin{matrix} 1, 1 \\ 0, 0, a t \end{matrix} \right)$$ and $$G_4 = G^{4,0}_{3,4}\left( \beta \bigg | \begin{matrix} 1, 1, 1 \\ 0, 0, 0, a t \end{matrix} \right)$$. Furthermore, $\psi ^{(1)}(l)$ is the trigamma function, i.e. the derivative of the digamma function $\psi ^{(0)}(l)$ with respect to *l*, and $\varGamma (l, z_1, z_2) = \int _{z_1}^{z_2} t^{l-1}e^{-t}dt$ is the generalised incomplete gamma function.

In our view, the best approach to deal with these functions is leveraging the approximation to the gamma cdf which was proposed by Moore (2018) and adapted by Yee in the R package VGAM (Yee 2010), which also includes the computation of first and second order derivatives (function pgamma.deriv()). In fact, the algorithm based on the above-mentioned equations in their exact formulation did not perform better than the approximation-based one, at the cost of a much longer training time (from the order of minutes to the order of hours). The approximation in Yee (2010) is obtained as a series expansion for $at \le \beta \le 1$ and $\beta < at$; otherwise, with a continued fraction expansion. More details and complete expressions for the derivatives, adapted from Moore (2018), can be found in “Appendix 1.7”. To relate this approximation to our case, it is sufficient to consider that the upper and lower incomplete gamma functions are linked by

21 $$\begin{aligned} \varGamma (\eta , \lambda ) + \gamma (\eta , \lambda ) = \varGamma (\eta ), \end{aligned}$$

with $\gamma (\eta , \lambda ) = P(\eta , \lambda ) \varGamma (\eta )$, where $P(\eta , \lambda )$ is the gamma cdf, the derivatives of which are provided by the aforementioned R package. Therefore we can write $\varGamma (\eta , \lambda ) = \varGamma (\eta ) - \varGamma (\eta )P(\eta , \lambda )$.

##### Approximation-based expressions

In order to use pgamma.deriv() from the VGAM package, we shall express the functions of interest in terms of derivatives of the upper incomplete gamma function, which in turn can be expressed in terms of the lower incomplete gamma function $\gamma (\alpha (t), \beta )$ via Eq. (21). For ease of interpretation, we substitute $h(\alpha (t), \beta ) = \varGamma (\alpha (t), \beta )$ and $g(\alpha (t)) = \varGamma (\alpha (t))$ to avoid having the same symbol for the upper incomplete gamma function and the gamma function, so that we can write

22 $$\begin{aligned} h(\alpha , \beta ) = g(\alpha ) - g(\alpha )P(\alpha , \beta ), \end{aligned}$$

where, for simplicity, we avoid specifying the time dependency of the shape parameter, and instead consider $\alpha = \alpha (t) = at$.

The derivatives are:

23 $$\begin{aligned}&\frac{\partial h(\alpha , \beta )}{\partial \alpha } = \frac{d g(\alpha )}{d \alpha } - \biggl ( \frac{d g(\alpha )}{d \alpha } P(\alpha , \beta ) + g(\alpha )\varvec{\frac{\partial P(\alpha , \beta )}{\partial \alpha }} \biggr ) \end{aligned}$$

24 $$\begin{aligned}&\frac{\partial ^2 h(\alpha , \beta )}{\partial \alpha ^2} = \frac{d^2 g(\alpha )}{d \alpha ^2} - \biggl ( \frac{d^2 g(\alpha )}{d \alpha ^2} P(\alpha , \beta ) + 2 \frac{d g(\alpha )}{d \alpha } \varvec{\frac{\partial P(\alpha , \beta )}{\partial \alpha }} + \nonumber \\&\hspace{7.8cm} g(\alpha ) \varvec{\frac{\partial ^2 P(\alpha , \beta )}{\partial \alpha ^2}} \biggr ) \end{aligned}$$

25 $$\begin{aligned}&\quad \frac{\partial h(\alpha , \beta )}{\partial \beta } = - g(\alpha )\varvec{\frac{\partial P(\alpha , \beta )}{\partial \beta }} \end{aligned}$$

26 $$\begin{aligned}&\frac{\partial ^2 h(\alpha , \beta )}{\partial \beta ^2} = - g(\alpha )\varvec{\frac{\partial ^2 P(\alpha , \beta )}{\partial \beta ^2}} \end{aligned}$$

27 $$\begin{aligned}&\frac{\partial ^2 h(\alpha , \beta )}{\partial \alpha \partial \beta } = - \frac{d g(\alpha )}{d \alpha } \varvec{\frac{\partial P(\alpha , \beta )}{\partial \beta }} - g(\alpha )\varvec{\frac{\partial ^2 P(\alpha , \beta )}{\partial \alpha \partial \beta }} \end{aligned}$$

where the first and second derivatives of the gamma function with respect to its argument are, respectively,

28 $$\begin{aligned} \frac{d g(\alpha )}{d \alpha } = g(\alpha ) \psi ^{(0)}(\alpha ) \end{aligned}$$

and

29 $$\begin{aligned} \frac{d^2 g(\alpha )}{d \alpha ^2} = g(\alpha ) (\psi ^{(0)}(\alpha )^2 + \psi ^{(1)}(\alpha )), \end{aligned}$$

while the derivatives of $P(\alpha , \beta )$, emphasised in bold, are provided by the VGAM package.

The FHT pdf is obtained by differentiating the FHT cdf with respect to time; hence, we can express it as

30 $$\begin{aligned} \begin{aligned} f_{T}(t; \alpha (t), \beta )&= \frac{\partial }{\partial t} \biggl ( \frac{h(\alpha (t), \beta )}{g(\alpha (t))} \biggr ) \\&= \frac{1}{g(\alpha )^2} \biggl [ \frac{d \alpha }{d t} \biggl ( g(\alpha ) \frac{\partial h(\alpha , \beta )}{\partial \alpha } - h(\alpha , \beta ) \frac{d g(\alpha )}{d \alpha } \biggr ) \biggr ], \end{aligned} \end{aligned}$$

where clearly $\frac{d \alpha }{d t}=a$. We then use Eqs. (23) and (28) to evaluate $\ln f_{T}(t; \alpha (t), \beta )$, needed for the loss function (15), in the case of uncensored observations, in the boosting algorithm.

The negative gradients of Eq. (2) are obtained as partial derivatives of $\ln f_{T}$ with respect to *a* and $\beta$, and we can formulate them as functions of equations (23) - (29):

31 $$\begin{aligned} \begin{aligned} u_a&= \frac{g(\alpha ) \biggl [ g(\alpha ) \left( \frac{\partial ^2 \alpha }{\partial a \partial t} \frac{\partial h(\alpha , \beta )}{\partial \alpha } + \frac{\partial \alpha }{\partial t} \frac{\partial \alpha }{\partial a} \frac{\partial ^2 h(\alpha , \beta )}{\partial \alpha ^2}\right) - 2 \frac{\partial \alpha }{\partial t} \frac{\partial \alpha }{\partial a} \frac{\partial h(\alpha , \beta )}{\partial \alpha } \frac{d g(\alpha )}{d \alpha } \biggr ]}{ \frac{\partial \alpha }{\partial t} \ g(\alpha ) \bigr ) \bigl ( g(\alpha ) \frac{\partial h(\alpha , \beta )}{\partial \alpha } - h(\alpha , \beta ) \frac{d g(\alpha )}{d \alpha }} \\ &\quad +\frac{h(\alpha , \beta ) \left( -\frac{\partial \alpha }{\partial t} \frac{\partial \alpha }{\partial a} g(\alpha ) \frac{d^2 g}{d \alpha ^2} + 2 \frac{\partial \alpha }{\partial t} \frac{\partial \alpha }{\partial a} \bigl ( \frac{d g(\alpha )}{d \alpha } \bigr )^2 - \frac{\partial ^2 \alpha }{\partial a \partial t} g(\alpha ) \frac{d g(\alpha )}{d \alpha } \right) }{ \frac{\partial \alpha }{\partial t} \ g(\alpha ) \bigr ) \bigl ( g(\alpha ) \frac{\partial h(\alpha , \beta )}{\partial \alpha } - h(\alpha , \beta ) \frac{d g(\alpha )}{d \alpha } } \end{aligned} \end{aligned}$$

and

32 $$\begin{aligned} \begin{aligned} u_b = \frac{\frac{\partial h(\alpha , \beta )}{\partial \beta } \frac{d g(\alpha )}{d \alpha } - g(\alpha ) \frac{\partial ^2 h(\alpha , \beta )}{\partial \alpha \partial \beta } }{h(\alpha , \beta )\frac{d g(\alpha )}{d \alpha } - g(\alpha ) \frac{\partial h(\alpha , \beta )}{\partial \alpha }}, \end{aligned} \end{aligned}$$

where $$\frac{\partial ^2 \alpha }{\partial a \partial t} = 1$$.

For censored observations, contributions to the loss function (15) come through the logarithm of the survival function, $\ln S_T$, where

33 $$\begin{aligned} S_T(t; \alpha , \beta ) = 1 - F_T(t; \alpha , \beta ) = 1 - \frac{h(\alpha , \beta )}{g(\alpha )}. \end{aligned}$$

The negative gradients are, in this case,

34 $$\begin{aligned} u_\alpha = - \frac{\frac{\partial \alpha }{\partial a} (h(\alpha , \beta ) \frac{d g(\alpha )}{d \alpha } - g(\alpha )\frac{\partial h(\alpha , \beta )}{\partial \alpha } )}{g(\alpha )(h(\alpha , \beta ) - g(\alpha ))} \end{aligned}$$

and

35 $$\begin{aligned} u_\beta = \frac{\frac{\partial h(\alpha , \beta )}{\partial \beta }}{h(\alpha , \beta )-g(\alpha )}. \end{aligned}$$

**Algorithm 2:**
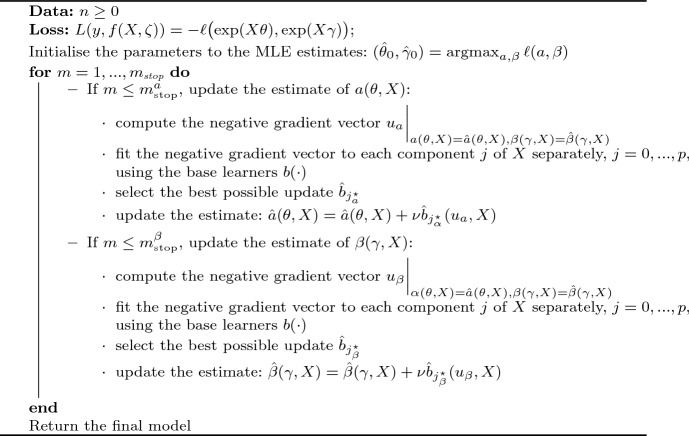
Boosted FHT based on a gamma process

## Results

### Simulated data

Before analysing real-world data from different applications, we explore the algorithm performance through simulated scenarios. The purpose of this section is to provide a simple demonstration of the algorithm using two examples with simulated data. However, to assess the method’s performance under various sample sizes and censoring proportions, a simulation study has been carried out and is provided as Online Resource. The supplementary material also includes an evaluation of the predictive performance of different models on the simulated data of the first example. The data for the examples and the simulation study were generated using a rejection sampling algorithm, described in “Appendix 1.8”. To focus the analysis and avoid unnecessary complexity, we have limited these simulations to the cyclic version of the algorithm. However, to provide a more comprehensive evaluation of the method, when applying it to real-world data we also explore its non-cyclic version.

#### Illustrative example 1

First, we generate $n=500$ data points from the FHT distribution originating from a gamma process with parameters

36 $$\begin{aligned} \begin{aligned}&\ln (a) = 1.5 + 3 x_1 - 1.5 x_2 \\&\ln (\beta ) = 2 + 0.25 x_3 - 0.5 x_4 \\&\, \, \text {with} \quad x_1, x_2, x_3, x_4 \overset{\text {iid}}{\sim } U(0, 1), \end{aligned} \end{aligned}$$

where *U*(0, 1) is the standard uniform distribution, and with censoring times taken independently from an exponential with rate 0.1, corresponding to a censoring proportion of about 10%. Table 1 displays the estimated parameters as the model is built step by step: we see that each iteration brings a small update, starting from the null model (at iteration 0, the initialisation step) and reaching a final model where all covariates are included. All the variables get an estimated coefficient which is reasonably close to the true value (shown in bold at the bottom of the table) at iteration 1000.

#### Illustrative example 2

As a further example, we reproduce a scenario with a larger number of covariates, including 0-effects. The true values for the parameters are as follows:

37 $$\begin{aligned} \begin{aligned}&\ln (a) = 2.5 + 0.3 \sum _{j=1}^{10} x_j + 0 \sum _{k=11}^{20} x_k \quad \quad \text {with} \, \, x_l \overset{\text {iid}}{\sim } U(0, 1) \quad \text {for} \, \, l=1,\ldots ,20 \\&\ln (\beta ) = 2.0 - 0.3 \sum _{j=1}^{10} z_j + 0 \sum _{k=11}^{20} z_k \, \, \, \quad \text {with} \quad z_l \overset{\text {iid}}{\sim } U(0, 1) \quad \text {for} \, \, \, l=1,\ldots ,20. \\ \end{aligned} \end{aligned}$$

We have generated $n=500$ observations with independent censoring times from an exponential with rate 3, leading to a censoring proportion of about 10%. The estimated parameters are presented in Table 2, only for the thousandth and two-thousandth iterations due to lack of space. In this case, in fact, the number of iterations has been doubled due to the presence of more covariates compared to the previous example. True values are displayed in bold at the bottom of the table.

The algorithm has successfully identified the intercepts and, with varying degrees of accuracy, the effect of $x_1,\ldots , x_8$ (note that, instead, $x_9$ and $x_{10}$ enter the model only at the two-thousandth observation), and $z_1,\ldots , z_{10}$: particularly in the case of the latter, sometimes a greater effect is attributed to some variables and a lesser effect to others, but the difference is reasonably low. Regarding the variables with zero coefficient, $\theta _{11}$, $\theta _{12}$, and $\theta _{13}$ are erroneously included in the model, but with extremely low coefficients; the same goes for $\gamma _{11}$ to $\gamma _{20}$, with the exception of $\gamma _{16}$, $\gamma _{17}$, and $\gamma _{18}$: $\gamma _{16}$ is assigned a coefficient of 0.2, whose effect is likely counterbalanced by the coefficients of $-\,0.15$ and $-\,0.1$ of $\gamma _{17}$ and $\gamma _{18}$. The inclusion of these spurious effects could probably have been avoided, at least partially, with an earlier stop; however, the overall performance of the model seems satisfactory.

**Table 1:** Parameters estimated with the boosted FHT model based on the gamma process for the first simulated example, as a function of the iteration number

Iter	${\ln \alpha }$			${\ln \beta }$		
	${\theta _0}$	${\theta _1}$	${\theta _2}$	${\gamma _0}$	${\gamma _1}$	${\gamma _2}$
1	− 0.096	0.277	–	0.838	–	–
2	− 0.096	0.520	–	0.811	–	–
3	− 0.096	0.732	–	0.783	–	–
4	− 0.096	0.921	–	0.755	–	–
5	− 0.096	0.921	− 0.178	0.727	–	–
6	− 0.096	1.091	− 0.178	0.699	–	–
7	− 0.096	1.091	− 0.336	0.672	–	–
8	− 0.096	1.247	− 0.336	0.645	–	–
9	− 0.096	1.247	− 0.476	0.620	–	–
10	− 0.096	1.340	− 0.476	0.594	–	–
50	− 0.054	2.515	− 1.409	0.286	0.154	− 0.392
100	0.072	2.664	− 1.508	0.383	0.231	− 0.488
200	0.319	2.690	− 1.515	0.703	0.231	− 0.488
300	0.506	2.709	− 1.522	0.936	0.231	− 0.488
400	0.651	2.724	− 1.533	1.110	0.231	− 0.488
500	0.765	2.735	− 1.541	1.244	0.231	− 0.488
600	0.856	2.745	− 1.547	1.351	0.231	− 0.488
700	0.931	2.752	− 1.549	1.436	0.231	− 0.486
800	0.991	2.759	− 1.554	1.504	0.231	− 0.479
900	1.040	2.764	− 1.555	1.560	0.231	− 0.473
1000	1.081	2.768	− 1.558	1.606	0.231	− 0.467
True	**1.500**	**3.000**	**− 1.500**	**2.000**	**0.250**	**− 0.500**

**Table 2:** Parameters estimated with the boosted FHT model based on the gamma process for a simulated sparser scenario at the thousandth and two-thousandth iteration

	$${\ln \alpha }$$								
Iter	${\theta _0}$	${\theta _1}$	${\theta _2}$	${\theta _3}$	${\theta _4}$	${\theta _5}$	${\theta _6}$	${\theta _7}$	${\theta _8}$
1000	2.420	0.198	0.286	0.106	0.151	0.118	0.080	0.167	0.155
2000	2.447	0.260	0.328	0.165	0.200	0.183	0.127	0.222	0.219
True	**2**.**500**	**0**.**300**	**0**.**300**	**0**.**300**	**0**.**300**	**0**.**300**	**0**.**300**	**0**.**300**	**0**.**300**
Iter	${\theta _9}$	${\theta _{10}}$	${\theta _{11}}$	${\theta _{12}}$	${\theta _{13}}$	${\theta _{14}}$	${\theta _{15}}$	${\theta _{16}}$	${\theta _{17}}$
1000	− 0.005	–	–	–	–	–	–	–	–
2000	0.007	0.009	− 0.020	− 0.010	− 0.052	–	–	–	–
True	**0**.**300**	**0**.**300**	**0**.**00**	**0**.**000**	**0**.**000**	**0**.**000**	**0**.**000**	**0**.**000**	**0**.**000**
Iter	${\theta _{18}}$	${\theta _{19}}$	${\theta _{20}}$						
1000	–	–	–						
2000	–	–	–						
True	**0**.**000**	**0**.**000**	**0**.**000**						

	$${\ln \beta }$$								
Iter	${\gamma _0}$	${\gamma _1}$	${\gamma _2}$	${\gamma _3}$	${\gamma _4}$	${\gamma _5}$	${\gamma _6}$	${\gamma _7}$	${\gamma _8}$
1000	1.881	− 0.305	− 0.282	− 0.406	− 0.295	− 0.203	− 0.364	− 0.332	− 0.363
2000	1.906	− 0.305	− 0.284	− 0.414	− 0.303	− 0.184	− 0.355	− 0.332	− 0.364
True	**2**.**000**	**− 0.300**	**− 0.300**	**− 0.300**	**− 0.300**	**− 0.300**	**− 0.300**	**− 0.300**	**− 0.300**
Iter	${\gamma _9}$	${\gamma _{10}}$	${\gamma _{11}}$	${\gamma _{12}}$	${\gamma _{13}}$	${\gamma _{14}}$	${\gamma _{15}}$	${\gamma _{16}}$	${\gamma _{17}}$
1000	− 0.381	− 0.422	− 0.012	0.090	− 0.029	− 0.074	0.223	− 0.154	− 0.115
2000	− 0.391	− 0.422	− 0.007	0.003	0.095	− 0.013	− 0.059	0.214	− 0.149
True	**− 0.300**	**− 0.300**	**0**.**000**	**0**.**000**	**0**.**000**	**0**.**000**	**0**.**000**	**0**.**000**	**0**.**000**
Iter	${\gamma _{18}}$	${\gamma _{19}}$	${\gamma _{20}}$						
1000	− 0.136	0.093	–						
2000	− 0.107	− 0.135	0.096						
True	**0**.**000**	**0**.**000**	**0**.**000**						

#### Simulation study (online resources)

Supplementary online resources provide additional details on our simulation study and predictive performance evaluations. In these simulations, 1000 datasets were generated with two different sample sizes (*n* = 100 and *n* = 500) and varying censoring levels (10%, 50%, and 90%) to assess how these factors affect the accuracy and variability of the parameter estimates. The results show that, as expected, larger sample sizes lead to more precise and robust estimates—as demonstrated by narrower, well-centered boxplots - while higher censoring rates introduce greater variability and bias, particularly in smaller samples. In addition, when comparing the two scenarios from the examples presented previously, the algorithm performed significantly better in cases with a strong underlying signal (Example 1) than in sparser conditions with many covariates of minimal effect (Example 2). Furthermore, predictive performance was assessed on the simulated data using integrated Brier scores and concordance indexes. The FHTgamma model, aligned with the data-generating mechanism, consistently demonstrates superior predictive performance compared to the other models under consideration.

### Real-world data

In this section we illustrate the performance of our algorithm in real data examples from both engineering (Sect. 3.2.1) and biomedical (Sects. 3.2.2, 3.2.3 and 3.2.4) applications. Note that the response variable exhibited a wide range with many values very close to zero in the last three cases, leading to numerical instabilities and convergence issues. To address these challenges, it was divided by a factor of 5 and shifted away from zero by adding 2. This transformation was designed to make the data more manageable without altering the fundamental form of its distribution. To have some reference for evaluating our method, we compare the following models:

- *boostFHT-Gamma*: Our boosted FHT model based on an underlying gamma process, with either stumps or linear base learners, as specified in each case. Results are shown both for the cyclic and noncyclic versions (abbreviated, respectively, FHTgamma_cyclic or simply FHTgamma and FHTgamma_noncyclic or FHTgamma_nc)
- *boostFHT-Wiener*: A boosted FHT model based on an underlying Wiener process with linear base learners, as in De Bin and Stikbakke (2022). Results are shown both for the cyclic and noncyclic versions (abbreviated, respectively, FHTwiener_cyclic or simply FHTwiener and FHTwiener_noncyclic or FHTwiener_nc)
- Cox: a Cox model (for the low-dimensional engineering application) or a boosting model with Cox loss (for the high-dimensional biomedical applications where the traditional Cox model would not work).

In addition, as a further reference we consider the Kaplan–Meier (KM) estimator. In all the examples we use 2/3 of the available data for training and 1/3 for testing. The boosting shrinkage parameter is set to the default value $\nu = 0.1$, while the number of boosting iterations $m_{\tiny {\hbox {stop}}}$ is chosen via fivefold cross validation.

The considered evaluation metrics are the following:

- Brier score (BS): a weighted average of the squared distances between the predicted survival probability and the observed survival status, where the weights are roughly the probabilities of the observations not being censored. For a proper definition and more details, we refer to Graf et al. (1999).
- Concordance index (C-index): a generalisation of the area under the ROC curve that accounts for censored observations. It evaluates the capability of the model to rank correctly the survival times of the individuals, based on their risk score. For its definition and more discussion, refer to Gerds et al. (2013).

Additionally, we provide aggregated error metrics obtained for each of 100 different repetitions (runs) of the analysis: in fact, De Bin and Stikbakke (2022) and many other authors warn not to overinterpret the results from a single run, as relying on a single train-test split can lead to biased results due to possible imbalance between the sets. Specifically, we show: the integrated Brier score (IBS), i.e. the result of integrating the Brier scores over the test set timespan; the truncated IBS, where Brier scores are integrated only up to a certain time $t^\star$ chosen *ad hoc* for each application; and the average C-index. The truncated IBS are provided since high censoring and sparse data can lead to less reliable and more variable estimates at later times, making the score less informative for assessing the models’ performance. All error metrics have been computed by means of the package pec (Mogensen et al. 2012) in R and are provided in the form of boxplots.

#### Toyota Research Institute lithium-ion batteries

The first application we consider is the degradation of lithium(Li)-ion batteries. Li-ion batteries constitute one of the most popular battery technologies and are a key factor of the “green shift” which ought to take place in many contexts nowadays. Estimation of the batteries’ Remaining Useful Life (RUL) is crucial to ensure safe operations and covering of power and energy demands. The End of Life (EoL) can be defined to be reached when the battery capacity, which falls during usage as a result of battery degradation, reaches 80% of the battery initial capacity (Vanem et al. 2021). In this case, the degradation itself is the underlying process, and we want to model the FHT of the threshold which is 80% of the nominal capacity. Since the degradation of the battery capacity is mostly monotonic, it seems appropriate to assume that the underlying process is a gamma process. Furthermore, as mentioned before, since the estimation of the battery capacity is a difficult task which in real life may be seldom performed—see e.g. Bertinelli Salucci et al. (2023) -, we treat the underlying process as unobservable.

The Li-ion battery dataset we consider is provided by the Toyota Research Institute—publicly available at (Toyota 2019) -, and consists of 125 lithium-ion phosphate (LFP)/graphite cells (nominal capacity of 1.1 Ah and nominal voltage of 3.3 V) cycled in lab under fast-charging policies. We refer to Severson et al. (2019) for a more detailed description of the dataset. The dataset has been processed to remove batteries with spurious data and missing values in the covariates, which are 11 variables extracted from the sensor data (data directly measured from battery operations, concerning voltage, current and temperature; definitions and formulas for the covariates can be found in “Appendix 1.9”, Table 4).

In the case of Li-ion batteries, it is not particularly meaningful to consider the variable of interest in terms of times (be it seconds, hours or days), as it would mean taking into account also the time during which the battery has not been used. Since our focus is on the battery ageing due to its usage (cycle ageing), we rather consider the amount of equivalent full cycles (EFC) the battery has undergone. Thus, the median follow-up “duration” of the experiment is 786 cycles, and the amount of censored observations in the dataset is 38.26%. Due to evident non-linearities between most covariates and the response, we have chosen stumps as base learners in our boosting algorithm.

**Fig. 1:**
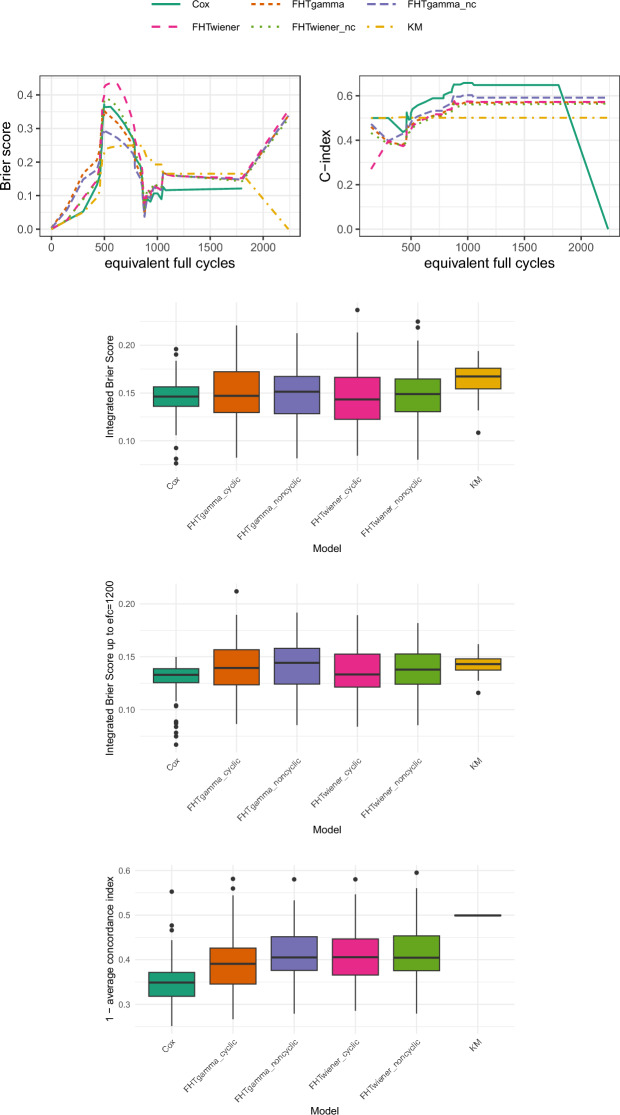
Results for the Li-Ion battery data. The top panels display the Brier scores (left) and the C-index (right) for the first of 100 runs. Below these, the boxplots show the integrated Brier scores over the entire range of EFC for 100 runs, the integrated Brier scores up to EFC = 1200 for 100 runs, and the distance between the average C-index of the 100 runs and the optimal C-index value of 1. In all boxplots, lower values indicate better performance, while in the top right plot (C-index), higher values correspond to better performance

Figure 1 shows the results we have gathered with respect to the performance metrics listed in Sect. 3.2. The two upper panels show the Brier scores and C-indexes for the first of the 100 runs (corresponding to set.seed(1)), while the three boxplots display the aggregated results for all runs. In the second boxplot, the integrated Brier score has been computed up to efc$=1200$. Looking at the single-run results, it is noteworthy that all the considered models perform worse than the KM estimator until approximately efc$=750$. For larger efc, the Cox model appears more appropriate than the FHT models, which are almost indistinguishable. The issues encountered in the first portion of the range are also evident in the C-index plot, where all models perform worse than random guessing until around efc$=750$. Subsequently, the performance of the various models improves, though remaining rather poor. The two versions of FHTwiener and the cyclic version of FHTgamma produce nearly identical results. A better performance is achieved with the non-cyclic version of FHTgamma, which is still outperformed by the Cox model, although the latter seems to struggle for efc$>1760$, both in relation to the C-index and the Brier score. Looking at the aggregated errors, it appears that all models improve compared to the KM estimator, with performance on average quite similar to each other. The cyclic version of FHTgamma seems to provide a modest improvement over the other FHT models in terms of the C-index, which, however, remains rather low for all models, indicating suboptimal performance.

#### Dutch breast cancer

As first biomedical application, following Bøvelstad et al. (2009) and De Bin and Stikbakke (2022) we consider the Dutch Breast Cancer (DBC) Dataset (Van’t Veer et al. 2002; Van De Vijver et al. 2002) in the version of Van Houwelingen et al. (2006) and fit the model using linear base learners. In this case, we have 295 (possibly censored) survival times pertaining to Dutch women affected by breast cancer; as covariates, the dataset includes 4919 gene expressions together with three clinical measurements (tumor diameter, lymph node status, and grade). The median observed time is 7.2 years, with a quite high censoring rate (73.2%).

**Fig. 2:**
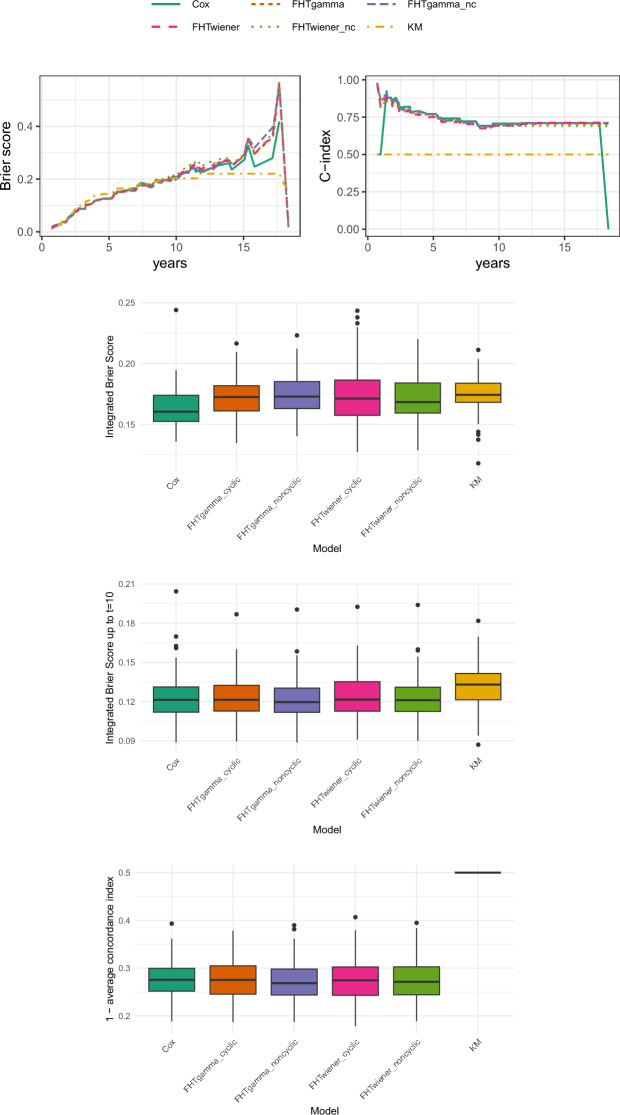
Results for the breast cancer data. The top panels display the Brier scores (left) and the C-index (right) for the first of 100 runs. Below these, the boxplots show the integrated Brier scores over the entire time range for 100 runs, the integrated Brier scores up to $t=10$ for 100 runs, and the distance between the average C-index of the 100 runs and the optimal C-index value of 1. In all boxplots, lower values indicate better performance, while in the top right plot (C-index), higher values correspond to better performance

Figure 2 presents our results following the same scheme as in Fig. 1. For the single run, we again find that the differences in performance between the models are very small, and again, worse than the KM estimator for half of the time range; the models perform better in terms of the C-index, though the Cox boosting model encounters issues at the extremes of the range. Looking at the aggregated results for 100 runs, all models are very close both in terms of IBS and average C-index. When considering the IBS, the Cox model is ranked first when integrating over the entire time span, but the difference is attenuated when integrating only up to $t=10$: in these case, all models appear almost equally good, with some improvement over the KM estimator.

#### Neuroblastoma

As a second biomedical application, again following De Bin and Stikbakke (2022), we use data about patients affected by neuroblastoma (NB) (Oberthuer et al. 2008) in the version of De Bin et al. (2014): the dataset contains 273 observations, $79\%$ of which are censored, with a median observation time of 3.8 years. We have again a mixture of molecular and clinical covariates, which puts us in a high-dimensional setting as in the previous example, and we choose again linear base learners for the boosting algorithm.

**Fig. 3:**
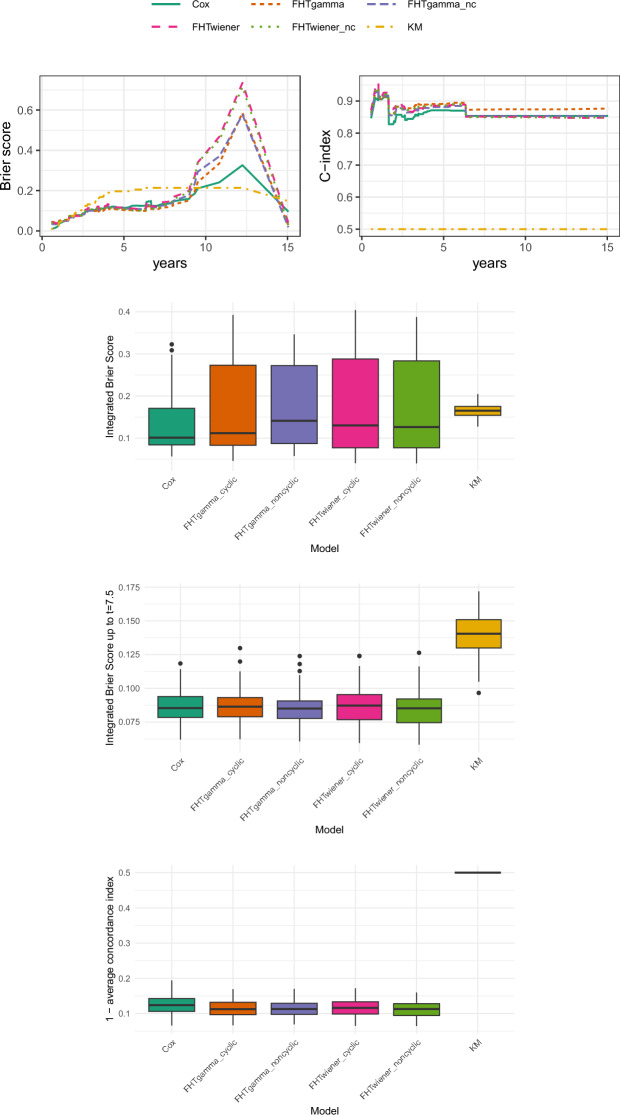
Results for the neuroblastoma data. The top panels display the Brier scores (left) and the C-index (right) for the first of 100 runs. Below these, the boxplots show the integrated Brier scores over the entire time range for 100 runs, the integrated Brier scores up to $t=7.5$ for 100 runs, and the distance between the average C-index of the 100 runs and the optimal C-index value of 1. In all boxplots, lower values indicate better performance, while in the top right plot (C-index), higher values correspond to better performance

Results are displayed in Fig. 3 for a single run and for 100 runs. In the single run, all models are quite close until $t=9$; from this point onward, the performance of the models suddenly worsens, shifting from being better than the KM estimator to being considerably worse. The C-index plots, instead, shows a quite good performance for all models, with the cyclic version of boostFHT-Gamma as the best and Cox as the worst, though by a small margin. Looking at the IBS over the entire time span, the performance of the FHT models is, on average, close to that of Cox and better than the KM estimator, but with much greater variability. The FHTgamma models seem to offer a small improvement over the FHTwiener models. This large variability disappears when considering the IBS only up to $t=7.5$, which leads us to believe that it was due to situations similar to those observed in the single run, with large errors in the final part of the time span. When it comes to the average C-index measures, all models achieved very good results, and in this case, the FHT models appear to be marginally better than Cox.

#### Diffuse large-B-cell lymphoma

**Fig. 4:**
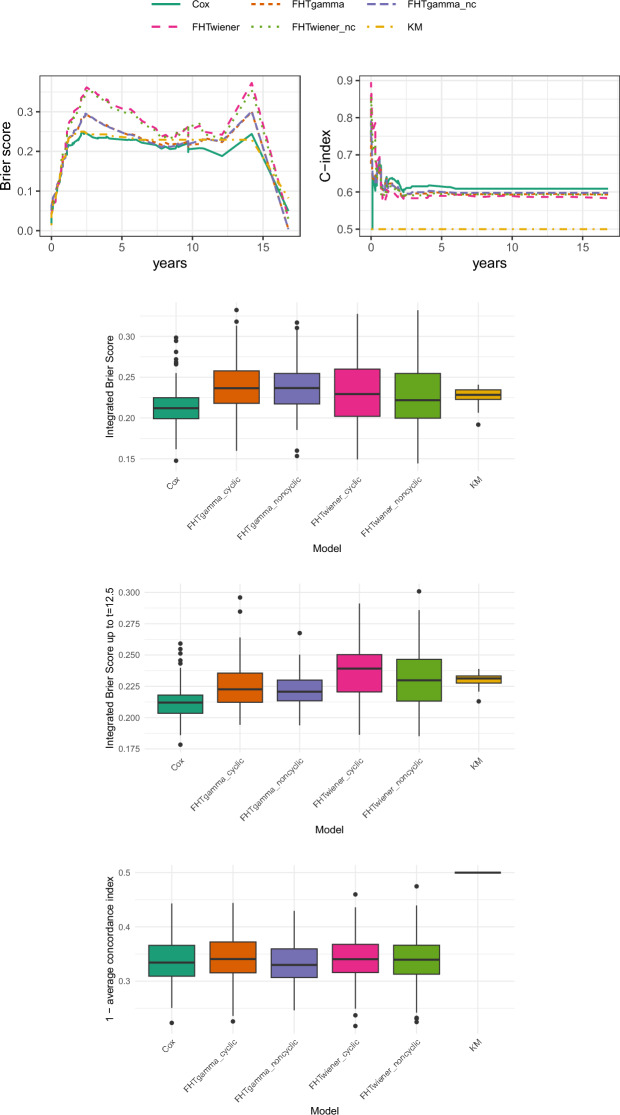
Results for the diffuse large-B-cell lymphoma data. The top panels display the Brier scores (left) and the C-index (right) for the first of 100 runs. Below these, the boxplots show the integrated Brier scores over the entire time range for 100 runs, the integrated Brier scores up to $t=12.5$ for 100 runs, and the distance between the average C-index of the 100 runs and the optimal C-index value of 1. In all boxplots, lower values indicate better performance, while in the top right plot (C-index), higher values correspond to better performance

Lastly, we consider the Diffuse Large-B-Cell Lymphoma (DLBCL) as in Bøvelstad et al. (2009) and Bøvelstad et al. (2009): here we have 222 observations with 43$\%$ of censoring, with a median observation time of 2.8 years. The covariates are constituted by 7399 molecular expressions, plus the International Prognostic Index expressed in three levels (low, medium, high), hence we adopt as before linear base learners for all boosting models. The results, shown in Fig. 4, do not look particularly promising for the single run, where the Cox model is the only model which performs slightly better than the KM estimator in terms of Brier scores, and the C-indexes are in general quite low with the Cox boosting model in the lead. The aggregated results for the integrated Brier scores on the whole range are somehow confirming this on average, but show that the FHT models can in fact be better than both KM and Cox, and the large variability suggests that the splitting between training and test data should be handled carefully. When integrating the Brier scores up to $t=12.5$, the picture changes significantly: the Cox model is confirmed to be the best option, but the FHT models based on a gamma process are on average better than those based on a Wiener process, and often improve over the KM estimator. Looking at the aggregated C-index results, all models look almost equivalent, but the noncyclic version of the FHTgamma has a lower median compared to the other models, including Cox.

### Training time

Table 3 compares the training times (given in minutes) for a single run^1^ of all models across the different datasets, including the simulated examples. In the case of the four application cases, the training time includes the cross-validation process. Note that the for the simulated scenarios, only the cyclic version of the FHTgamma boosting model has been run, therefore the training times of the other models are missing. The results reveal significant variations in computational efficiency. FHTgamma generally requires the most time, due to the complexity of the model, while the Cox model is consistently the fastest to train.

**Table 3:** Training time for the considered models (min)

	Example 1	Example 2	Li-ion	DBC	NB	DLBCL
FHTgamma	0.3	0.7	1.6	3.6	6.5	3.6
FHTgamma_nc	–	–	0.35	1.3	3.8	2.3
FHTwiener	–	–	0.5	1.4	3.8	2.1
FHTwiener_nc	–	–	0.2	1.4	4.3	2.3
Cox	–	–	0.01	0.2	0.4	0.2

Note: for the application cases (Li-ion, DBC, NB, DLBCL) the provided timing includes the cross validation process

## Conclusions

This paper presented a boosting algorithm for fitting a first hitting time model based on an underlying homogeneous gamma process, specifically conceived for degradation processes where no recovery effect is expected. First hitting time models can be considered an alternative to the Cox model in situations where the proportional hazards assumption does not hold or cannot be tested, with the advantage of modelling the response (time-to-event) directly. However, the necessity of choosing a parametric model beforehand can limit the flexibility of the method compared to the Cox regression model. Diagnostics to support the choice of model after it has been fitted should focus on assessing both the adequacy of the chosen parametric form and the predictive performance of the model. In the context of this article, we have focused on the latter, utilising Brier scores and C-indexes to compare the performance of our model against the Kaplan–Meier estimator and other models. Another potential diagnostic could have involved verifying the distribution of Cox-Snell residuals to assess the model’s overall fit.

Our method has showed good results both on simulated data and on data from different application cases. The analysis on simulated scenarios demonstrates that the algorithm performs robustly in dense settings with strong signals, even under high censoring conditions. However, the performance can decline in sparser scenarios with many covariates and weaker signals, particularly with smaller sample sizes and higher censoring rates. Increasing the sample size proves crucial in counteracting the increased variability and bias introduced by these challenging conditions. The real-data examples have been selected from different application areas to reflect different situations: note the low-dimensional scenario of Li-ion batteries versus the high-dimensionality of the biomedical applications, as well as the different percentage of censored observations (respectively $38\%, 73\%$, $79\%$ and $43\%$).

A minor trade-off of our algorithm is a longer training time, which, while noticeable, remains well within an acceptable range (from a few seconds to a few minutes). Another disadvantage, typical of boosting and other machine learning algorithms, is that we obtain point estimates for the parameters but lack estimates for their standard errors, which hinders the process of quantifying the uncertainty and variability associated with the model predictions. To address this issue, bootstrapping can be considered as a practical solution: by repeatedly fitting the boosting model to resampled subsets of the data, bootstrapping generates distributions of parameter estimates that can be used to calculate their standard errors.

In this article, heterogeneity among individuals has been introduced solely by regressing the underlying model parameters on the covariate matrix. We recommend exploring greater variability, such as incorporating random thresholds or individual random effects, in future works. Another promising area for future research could be extending this methodology to recurrent events, which may enhance the applicability and robustness of the first hitting time model for monotonic degradation in more complex settings.

What inspired this work was the aim to create a suitable model for non-decreasing processes. In the case of batteries, it is well-established that capacity does not improve over time, unless for varied conditions or extended resting periods. In the context of personal health, using a first hitting time model based on a gamma process rather than a Wiener process involves a different philosophical approach. In the latter, as in De Bin and Stikbakke (2022), an individual’s health is perceived as something that can fluctuate up and down, though decreasing in the long term; in this paper, we rather address the irreversible nature of the ageing process and underscore the fact that no one is getting younger by the day. Generally speaking, a Wiener process is ideal for systems characterised by continuous changes over time, with normally distributed, independent increments that lead to smooth changes and continuous paths. On the other hand, a gamma process is more appropriate for modeling situations that require non-negative, monotonic paths, with independent but not normally distributed increments, and are more suitable for data following a skewed distribution, or in cases where events may occur in discrete jumps or increments. Overall, it is worth emphasising that when prior knowledge about the degradation process is available, and the process is understood to be monotonic, leveraging this knowledge is advisable and is expected to lead to enhanced estimation.

## Supplementary Information

Below is the link to the electronic supplementary material.

Supplementary file 1 (pdf 384 KB)

## Appendix 1

### Appendix 1.1: Proof of Eq. (9)

$$\begin{aligned} f_{T}(t; a, \beta ) = \frac{a \biggl [ G^{3,0}_{2,3} \left( \beta \, \bigg | \begin{matrix} 1, 1 \\ 0, 0, a t \end{matrix} \right) + \varGamma (at, \beta )(\ln (\beta ) - \psi ^{(0)}(at)) \biggr ]}{\varGamma (at)} \end{aligned}$$

#### Proof

$$\begin{aligned} f_{T}(t; a, \beta )&= \frac{\partial }{\partial t} F_{T}(t; a, \beta ) = \frac{\partial }{\partial t} \left( \frac{\varGamma (at, \beta )}{\varGamma (at)} \right) \\ &= \frac{1}{\varGamma (at)}\frac{\partial \varGamma (at, \beta )}{\partial t} + \varGamma (at, \beta ) \frac{\partial }{\partial t}\bigl (\varGamma (at)^{-1}\bigr )\\ &= \frac{1}{\varGamma (at)}\frac{\partial \varGamma (at, \beta )}{\partial t} - \frac{a\varGamma (at, \beta )\varGamma (at)\psi ^{(0)}(at)}{\varGamma (at)^{2}} \\&= \frac{\varGamma (at) \frac{\partial \varGamma (at, \beta )}{\partial t} - a\varGamma (at, \beta )\varGamma (at)\psi ^{(0)}(at)}{\varGamma (at)^2} \end{aligned}$$

where we have used

$$\begin{aligned} \frac{\partial \varGamma (at)}{\partial t} = \frac{\partial \varGamma (at)}{\partial (at)} \frac{\partial (at)}{\partial t} = a \varGamma (at) \psi ^{(0)}(at). \end{aligned}$$

The derivative of the incomplete gamma function with respect to time is

$$\begin{aligned} \frac{\partial \varGamma (at, \beta )}{\partial t} = \frac{\partial \varGamma (at, \beta )}{\partial (at)} \frac{\partial (at)}{\partial t}. \end{aligned}$$

Furthermore, following Geddes et al. (1990),

$$\begin{aligned} \frac{\partial \varGamma (at, \beta )}{\partial (at)} = \ln \beta \varGamma (\alpha , \beta ) + \beta G^{3,0}_{2,3} \left( \beta \, \bigg | \begin{matrix} 0, 0 \\ -1, at-1, -1 \end{matrix} \right) \end{aligned}$$

with—see e.g. Bateman and Erdélyi (1955)

$$\begin{aligned} \beta G^{3,0}_{2,3} \left( \beta \, \bigg | \begin{matrix} 0, 0 \\ -1, at-1, -1 \end{matrix} \right) = G^{3,0}_{2,3} \left( \beta \, \bigg | \begin{matrix} 1, 1 \\ 0, at, 0 \end{matrix} \right) \end{aligned}$$

and, by definition as from Eq. (10),

$$\begin{aligned} G^{3,0}_{2,3} \left( \beta \, \bigg | \begin{matrix} 1, 1 \\ 0, at, 0 \end{matrix} \right) = G^{3,0}_{2,3} \left( \beta \, \bigg | \begin{matrix} 1, 1 \\ 0, 0, at \end{matrix} \right) . \end{aligned}$$

Therefore

$$\begin{aligned} \frac{\partial \varGamma (at, \beta )}{\partial (at)} = a \left[ \ln \beta \varGamma (\alpha , \beta ) + \beta G^{3,0}_{2,3} \left( \beta \, \bigg | \begin{matrix} 1, 1 \\ 0, 0, at \end{matrix} \right) \right] \end{aligned}$$

and

$$\begin{aligned} f_{T}(t; a, \beta )&= \frac{a\biggl [\varGamma (at, \beta ) \ln (\beta ) + G^{3,0}_{2,3} \left( \beta \, \bigg | \begin{matrix} 1, 1 \\ 0, 0, a t \end{matrix} \right) \biggr ] - a\varGamma (at, \beta ) \psi ^{(0)}(at)}{\varGamma (at)} \\&= \frac{a\biggl [\varGamma (at, \beta ) \ln (\beta ) + G^{3,0}_{2,3} \left( \beta \, \bigg | \begin{matrix} 1, 1 \\ 0, 0, a t \end{matrix} \right) - \varGamma (at, \beta ) \psi ^{(0)}(at)\biggr ]}{\varGamma (at)} \\ &= \frac{a\biggl [G^{3,0}_{2,3} \left( \beta \, \bigg | \begin{matrix} 1, 1 \\ 0, 0, a t \end{matrix} \right) + \varGamma (at, \beta ) [\ln (\beta ) - \psi ^{(0)}(at)]\biggr ]}{\varGamma (at)} \quad \end{aligned}$$

$$\square$$

### Appendix 1.2: Proof of Eq. (19)

$$\begin{aligned} u_a = \frac{ \left( \begin{aligned}&-\left( at \psi ^{(0)}(at) + 1\right) \left( G_3 + \varGamma (at, \beta )\left( \ln \beta - \psi ^{(0)}(at)\right) \right) \\ &\quad + at\left( 2G_4 + \ln \beta G_3\right) \\ &\quad + at\left( \ln \beta - \psi ^{(0)}(at)\right) \left( G_3 + \ln \beta \varGamma (at, \beta )\right) \\&\quad - at \psi ^{(1)}(at)\varGamma (at, \beta ) \end{aligned} \right) }{ a\left( G_3 + \varGamma (at, \beta )\left( \ln \beta - \psi ^{(0)}(at) \right) \right) } \end{aligned}$$

where $$G_3 = G^{3,0}_{2,3} \left( \beta \, \bigg | \begin{matrix} 1, 1 \\ 0, 0, a t \end{matrix} \right)$$ and $$G_4 = G^{4,0}_{3,4}\left( \beta \bigg | \begin{matrix} 1, 1, 1 \\ 0, 0, 0, a t \end{matrix} \right)$$.

#### Proof

$$\begin{aligned} u_a&= \frac{\partial }{\partial a} \ln f_{T}(t) = \frac{\partial }{\partial a} \ln \left( \frac{a \left( G_3 + \varGamma (a t, \beta ) \left( \ln \beta - \psi ^{(0)}(a t) \right) \right) }{\varGamma (a t)} \right) \\ &= \frac{\partial }{\partial a} \ln \left( a \left( G_3 + \varGamma (a t, \beta ) \left( \ln \beta - \psi ^{(0)}(a t) \right) \right) \right) - \ln (\varGamma (a t)) \\ &= -\left( \frac{\partial }{\partial a} \ln (\varGamma (a t)) \right) + \frac{\partial }{\partial a} \ln \left( a \left( G_3 + \varGamma (a t, \beta ) \left( \ln \beta - \psi ^{(0)}(a t) \right) \right) \right) \\&= -\left( \frac{t\varGamma (at) \psi ^{(0)}(at)}{\varGamma (a t)} \right) + \frac{\partial }{\partial a} \ln \left( a \left( G_3 + \varGamma (a t, \beta ) \left( \ln \beta - \psi ^{(0)}(a t) \right) \right) \right) \\&= -\left( t \psi ^{(0)}(at)\right) + \frac{\partial }{\partial a} \ln a + \frac{\partial }{\partial a} \ln \bigl ( G_3 + \varGamma (a t, \beta ) \left( \ln \beta - \psi ^{(0)}(a t) \right) \bigr ) \\ &= -\left( t \psi ^{(0)}(at)\right) + \frac{1}{a} + \frac{\left. \frac{\partial }{\partial a} \left( G_3 + \varGamma (a t, \beta ) \left( \ln \beta - \psi ^{(0)}(a t) \right) \right) \right. }{\biggl ( G_3 + \varGamma (a t, \beta ) \left( \ln \beta - \psi ^{(0)}(a t) \right) \biggr )} \\ &= -\left( t \psi ^{(0)}(at)\right) + \frac{1}{a} + \frac{\left. \left( \frac{\partial }{\partial a} G_3 + \frac{\partial }{\partial a}\left( \varGamma (a t, \beta ) \ln \beta \right) - \frac{\partial }{\partial a} \left( \varGamma (a t, \beta )\psi ^{(0)}(a t) \right) \right) \right. }{\biggl ( G_3 + \varGamma (a t, \beta ) \left( \ln \beta - \psi ^{(0)}(a t) \right) \biggr )} \\ \end{aligned}$$

where

- $\frac{\partial }{\partial a} G_3 = t \left( 2 G_4 + \ln \beta \, G_3 \right)$, see e.g. Gradshteyn and Ryzhik (2007) and Wolfram (2024)
- $\frac{\partial }{\partial a} \varGamma (at, \beta ) = t \left( G_3 + \ln (\beta ) \varGamma (a, \beta ) \right)$, see Geddes et al. (1990)
- $$\frac{\partial }{\partial a} \psi ^{0}(at) = t \psi ^{(1)}(at)$$

giving

$$\begin{aligned} u_a&= -\left( t \psi ^{(0)}(at)\right) + \frac{1}{a} + \frac{1}{\biggl ( G_3 + \varGamma (a t, \beta ) \left( \ln \beta - \psi ^{(0)}(a t) \right) \biggr )} \biggl [ t \left( 2 G_4 + \ln \beta \, G_3 \right) + \\ &\quad \quad t \ln \beta \biggl ( G_3 + \ln \beta \, \varGamma (at, \beta ) \biggr ) - \biggl ( t \psi ^{(0)}(at) \, G_3 + t \varGamma (at, \beta ) (\ln \beta \psi ^{(0)}(at) + \psi ^{(1)}(at)) \biggr ) \biggr ] \end{aligned}$$

or, taking the least common multiple of the denominator,

$$\begin{aligned} u_a = \frac{ \left( \begin{aligned}&-at\psi ^{(0)}(at)\left( G_3 + \varGamma (at, \beta )\left( \ln \beta - \psi ^{(0)}(at) \right) \right) + \biggl ( G_3 + \varGamma (at, \beta ) \\&\left( \ln \beta - \psi ^{(0)}(at) \right) \biggr ) + a\biggl [ t \left( 2 G_4 + \ln \beta \, G_3 \right) + t \ln \beta \biggl ( G_3 + \ln \beta \, \varGamma (at, \beta ) \biggr ) - \\ &\biggl ( t \psi ^{(0)}(at) \, G_3 + t \varGamma (at, \beta ) (\ln \beta \psi ^{(0)}(at) + \psi ^{(1)}(at)) \biggr ) \biggr ]\\ \end{aligned} \right) }{ a\left( G_3 + \varGamma (at, \beta )\left( \ln \beta - \psi ^{(0)}(at) \right) \right) } \end{aligned}$$

which is the same as

$$\begin{aligned} u_a = \frac{ \left( \begin{aligned}&-\left( at \psi ^{(0)}(at) + 1\right) \left( G_3 + \varGamma (at, \beta ) \left( \ln \beta - \psi ^{(0)}(at)\right) \right) \\&\quad + at\left( 2G_4 + \ln \beta G_3\right) \\&\quad + at\left( \ln \beta - \psi ^{(0)}(at)\right) \left( G_3 + \ln \beta \varGamma (at, \beta )\right) \\&\quad - at \psi ^{(1)}(at)\varGamma (at, \beta ) \end{aligned} \right) }{ a\left( G_3 + \varGamma (at, \beta )\left( \ln \beta - \psi ^{(0)}(at) \right) \right) } \end{aligned}$$

$$\square$$

### Appendix 1.3: Proof of Eq. (20)

$$\begin{aligned} u_\beta = \frac{-e^{-\beta } \beta ^{at-1} (\ln \beta - \psi ^{(0)}(at)) + \frac{\varGamma (at, \beta )}{\beta } - \frac{\pi \bigl ( \frac{\csc (\pi at)}{\varGamma (1-at)} - \frac{at \csc {\pi at} \varGamma (at, 0, \beta )}{\varGamma (1-at)\varGamma (1+at)} \bigr )}{\beta }}{G_3 + \varGamma (at, \beta )(\ln \beta - \psi ^{(0)}(at))}, \end{aligned}$$

with $$G_3 = G^{3,0}_{2,3} \left( \beta \, \bigg | \begin{matrix} 1, 1 \\ 0, 0, a t \end{matrix} \right)$$.

#### Proof

$$\begin{aligned} u_\beta&= \frac{\partial }{\partial \beta } \ln f_{T}(t; a, \beta ) = \frac{\partial }{\partial \beta } \ln \left( \frac{a \left( G_3 + \varGamma (a t, \beta ) \left( \ln \beta - \psi ^{(0)}(a t) \right) \right) }{\varGamma (a t)} \right) \\ &= \frac{\partial }{\partial \beta } \left( \frac{a \left( G_3 + \varGamma (a t, \beta ) \left( \ln \beta - \psi ^{(0)}(a t) \right) \right) }{\varGamma (a t)} \right) \frac{\varGamma (at)}{a \left( G_3 + \varGamma (a t, \beta ) \left( \ln \beta - \psi ^{(0)}(a t) \right) \right) } \\ &= \frac{ \frac{\partial }{\partial \beta } G_3 + \frac{\partial }{\partial \beta } \left( \varGamma (a t, \beta ) \ln \beta \right) - \frac{\partial }{\partial \beta } \left( \varGamma (at, \beta ) \psi ^{(0)}(a t) \right) }{ G_3 + \varGamma (a t, \beta ) \left( \ln \beta - \psi ^{(0)}(a t) \right) }. \end{aligned}$$

We have (Wolfram 2024)

$$\begin{aligned} \frac{\partial }{\partial \beta } \left( G^{3,0}_{2,3} \left( \beta \, \bigg | \begin{matrix} 1, 1 \\ 0, 0, a t \end{matrix} \right) \right) = \frac{\pi \csc (\pi at) \left( at \varGamma (at, 0, \beta ) - \varGamma (at + 1) \right) }{\beta \varGamma (1 - at) \varGamma (at + 1)}; \end{aligned}$$

furthermore,

$$\begin{aligned} \frac{\partial }{\partial \beta } \left( \varGamma (at, \beta ) \ln \beta \right)&= \ln \beta \, \frac{\partial }{\partial \beta } \varGamma (at, \beta ) + \varGamma (at, \beta ) \frac{\partial }{\partial \beta } \ln \beta = \ln \beta (-e^{-\beta }\beta ^{-1+at}) + \frac{\varGamma (at, \beta )}{\beta } \end{aligned}$$

and

$$\begin{aligned} \frac{\partial }{\partial \beta } \left( \varGamma (at, \beta ) \psi ^{(0)}(at) \right) = (-e^{-\beta }\beta ^{-1+at})\psi ^{(0)}(at). \end{aligned}$$

Hence

$$\begin{aligned} u_\beta&= \frac{ \left( \frac{\pi \csc (\pi at) \left( at \varGamma (at, 0, \beta ) - \varGamma (at + 1) \right) }{\beta \varGamma (1 - at) \varGamma (at + 1)} \right) - \ln \beta (e^{-\beta }\beta ^{-1+at}) + \frac{\varGamma (at, \beta )}{\beta } + e^{-\beta }\beta ^{-1+at}\psi ^{(0)}(at) }{ G_3 + \varGamma (a t, \beta ) \left( \ln \beta - \psi ^{(0)}(a t) \right) } \\ &= \frac{-e^{-\beta } \beta ^{at-1} (\ln \beta - \psi ^{(0)}(at)) + \frac{\varGamma (at, \beta )}{\beta } - \frac{\pi \bigl ( \frac{\csc (\pi at)}{\varGamma (1-at)} - \frac{at \csc {\pi at} \varGamma (at, 0, \beta )}{\varGamma (1-at)\varGamma (1+at)} \bigr )}{\beta }}{G_3 + \varGamma (at, \beta )(\ln \beta - \psi ^{(0)}(at))} \end{aligned}$$

$$\square$$

### Appendix 1.4: Proof of Eq. (31)

$$\begin{aligned} \begin{aligned} u_a&= \frac{g(\alpha ) \biggl [ g(\alpha ) \left( \frac{\partial ^2 \alpha }{\partial a \partial t} \frac{\partial h(\alpha , \beta )}{\partial \alpha } + \frac{\partial \alpha }{\partial t} \frac{\partial \alpha }{\partial a} \frac{\partial ^2 h(\alpha , \beta )}{\partial \alpha ^2}\right) - 2 \frac{\partial \alpha }{\partial t} \frac{\partial \alpha }{\partial a} \frac{\partial h(\alpha , \beta )}{\partial \alpha } \frac{d g(\alpha )}{d \alpha } \biggr ]}{ \frac{\partial \alpha }{\partial t} \ g(\alpha ) \bigr ) \bigl ( g(\alpha ) \frac{\partial h(\alpha , \beta )}{\partial \alpha } - h(\alpha , \beta ) \frac{d g(\alpha )}{d \alpha }} \\ &\quad + \frac{h(\alpha , \beta ) \left( -\frac{\partial \alpha }{\partial t} \frac{\partial \alpha }{\partial a} g(\alpha ) \frac{d^2 g}{d \alpha ^2} + 2 \frac{\partial \alpha }{\partial t} \frac{\partial \alpha }{\partial a} \bigl ( \frac{d g(\alpha )}{d \alpha } \bigr )^2 - \frac{\partial ^2 \alpha }{\partial a \partial t} g(\alpha ) \frac{d g(\alpha )}{d \alpha } \right) }{ \frac{\partial \alpha }{\partial t} \ g(\alpha ) \bigr ) \bigl ( g(\alpha ) \frac{\partial h(\alpha , \beta )}{\partial \alpha } - h(\alpha , \beta ) \frac{d g(\alpha )}{d \alpha } } \end{aligned} \end{aligned}$$

#### Proof

$$\begin{aligned} u_a&= \frac{\partial }{\partial a} \ln f_T(t; a, \beta ) = \frac{\partial }{\partial a} \left( \ln \left( \frac{1}{g(\alpha )^2} \biggl [ \frac{\partial \alpha }{\partial t} \biggl ( g(\alpha ) \frac{\partial h(\alpha , \beta )}{\partial \alpha } - h(\alpha , \beta ) \frac{d g(\alpha )}{d \alpha } \biggr ) \biggr ] \right) \right) \\ &= \frac{\partial }{\partial a} \left( \ln \left( \frac{\frac{\partial \alpha }{\partial t} \frac{\partial f(\alpha ,\beta )}{\partial \alpha }}{g(\alpha )} - \frac{\frac{\partial \alpha }{\partial t}h(\alpha , \beta ) \frac{dg(\alpha )}{d(\alpha )}}{g(\alpha )^2} \right) \right) \\ &= \frac{\frac{\partial }{\partial a}\left( \frac{\partial \alpha }{\partial t} \frac{\partial f(\alpha ,\beta )}{\partial \alpha } g(\alpha )^{-1} - \frac{\partial \alpha }{\partial t} h(\alpha , \beta ) \frac{dg(\alpha )}{d\alpha }g(\alpha )^{-2} \right) }{\left( \frac{\frac{\partial \alpha }{\partial t} \frac{\partial f(\alpha ,\beta )}{\partial \alpha }}{g(\alpha )} - \frac{\frac{\partial \alpha }{\partial t}h(\alpha , \beta ) \frac{dg(\alpha )}{d\alpha }}{g(\alpha )^2} \right) } \\ &= \frac{\frac{\partial }{\partial a} \left( \frac{\partial \alpha }{\partial t} \frac{\partial f(\alpha ,\beta )}{\partial (\alpha )} g(\alpha )^{-1} \right) - \frac{\partial \alpha }{\partial t} \left[ \frac{\partial }{\partial a} \left( h(\alpha , \beta )\frac{dg(\alpha )}{d\alpha }g(\alpha )^{-2}\right) \right] - h(\alpha , \beta )\frac{dg(\alpha )}{d\alpha }g(\alpha )^{-2}\frac{\partial ^2 \alpha }{\partial a \partial t}}{\left( \frac{\frac{\partial \alpha }{\partial t} \frac{\partial f(\alpha ,\beta )}{\partial \alpha }}{g(\alpha )} - \frac{\frac{\partial \alpha }{\partial t}h(\alpha , \beta ) \frac{dg(\alpha )}{d\alpha }}{g(\alpha )^2} \right) } \\ &= \frac{\frac{\partial }{\partial a} \left( \frac{\partial \alpha }{\partial t} \frac{\partial f(\alpha ,\beta )}{\partial \alpha } g(\alpha )^{-1} \right) - \frac{\partial \alpha }{\partial t} \left[ h(\alpha , \beta ) \frac{\partial }{\partial a} \left( \frac{dg(\alpha )}{d\alpha }g(\alpha )^{-2} \right) + \frac{\partial h(\alpha , \beta )}{\partial \alpha } \frac{\partial \alpha }{\partial a} \frac{dg(\alpha )}{d\alpha } g(\alpha )^{-2} \right] }{\left( \frac{\frac{\partial \alpha }{\partial t} \frac{\partial f(\alpha ,\beta )}{\partial \alpha }}{g(\alpha )} - \frac{\frac{\partial \alpha }{\partial t}h(\alpha , \beta ) \frac{dg(\alpha )}{d\alpha }}{g(\alpha )^2} \right) } \\ &\quad + \frac{-h(\alpha , \beta )\frac{dg(\alpha )}{d\alpha }g(\alpha )^{-2}\frac{\partial ^2 \alpha }{\partial a \partial t}}{\left( \frac{\frac{\partial \alpha }{\partial t} \frac{\partial f(\alpha ,\beta )}{\partial \alpha }}{g(\alpha )} - \frac{\frac{\partial \alpha }{\partial t}h(\alpha , \beta ) \frac{dg(\alpha )}{d\alpha }}{g(\alpha )^2} \right) } \\ &= \frac{ \frac{\partial ^2 \alpha }{\partial a\partial t} \frac{\partial f(\alpha ,\beta )}{\partial \alpha } g(\alpha )^{-1} + \frac{\partial \alpha }{\partial t} \frac{\partial }{\partial a} \left( \frac{\partial f(\alpha ,\beta )}{\partial \alpha } g(\alpha )^{-1} \right) }{\left( \frac{\frac{\partial \alpha }{\partial t} \frac{\partial f(\alpha ,\beta )}{\partial \alpha }}{g(\alpha )} - \frac{\frac{\partial \alpha }{\partial t}h(\alpha , \beta ) \frac{dg(\alpha )}{d\alpha }}{g(\alpha )^2} \right) } \\ &\quad + \frac{ -\frac{\partial \alpha }{\partial t} \left[ h(\alpha , \beta ) \left( \frac{\partial }{\partial a} \left( \frac{dg(\alpha )}{d\alpha } \right) g(\alpha )^{-2} + \frac{dg(\alpha )}{d\alpha } \frac{\partial }{\partial a} (g(\alpha )^{-2}) \right) + \frac{\partial h(\alpha , \beta )}{\partial \alpha } \frac{\partial \alpha }{\partial a} \frac{dg(\alpha )}{d\alpha } g(\alpha )^{-2} \right] }{\left( \frac{\frac{\partial \alpha }{\partial t} \frac{\partial f(\alpha ,\beta )}{\partial \alpha }}{g(\alpha )} - \frac{\frac{\partial \alpha }{\partial t}h(\alpha , \beta ) \frac{dg(\alpha )}{d\alpha }}{g(\alpha )^2} \right) } \\ &\quad + \frac{- h(\alpha , \beta )\frac{dg(\alpha )}{d\alpha }g(\alpha )^{-2}\frac{\partial ^2 \alpha }{\partial a \partial t}}{\left( \frac{\frac{\partial \alpha }{\partial t} \frac{\partial f(\alpha ,\beta )}{\partial \alpha }}{g(\alpha )} - \frac{\frac{\partial \alpha }{\partial t}h(\alpha , \beta ) \frac{dg(\alpha )}{d\alpha }}{g(\alpha )^2} \right) } \\ &= \frac{ \frac{\partial ^2 \alpha }{\partial a\partial t} \frac{\partial f(\alpha ,\beta )}{\partial \alpha } g(\alpha )^{-1} + \frac{\partial \alpha }{\partial t} \left( \frac{\partial ^2 f(\alpha ,\beta )}{\partial \alpha ^2} g(\alpha )^{-1} + \frac{\partial f(\alpha ,\beta )}{\partial \alpha }\frac{\partial }{\partial a} \left( g(\alpha )^{-1} \right) \right) }{\left( \frac{\frac{\partial \alpha }{\partial t} \frac{\partial f(\alpha ,\beta )}{\partial \alpha }}{g(\alpha )} - \frac{\frac{\partial \alpha }{\partial t}h(\alpha , \beta ) \frac{dg(\alpha )}{d\alpha }}{g(\alpha )^2} \right) } \\ &\quad + \frac{ -\frac{\partial \alpha }{\partial t} \left[ h(\alpha , \beta ) \left( \frac{\partial \alpha }{\partial a} \frac{d^2 g(\alpha )}{d\alpha ^2} g(\alpha )^{-2} - 2 \left( \frac{dg(\alpha )}{d\alpha } \right) ^2 g(\alpha )^{-3} \frac{\partial \alpha }{\partial a} \right) + \frac{\partial h(\alpha , \beta )}{\partial \alpha } \frac{\partial \alpha }{\partial a} \frac{dg(\alpha )}{d\alpha } g(\alpha )^{-2} \right] }{\left( \frac{\frac{\partial \alpha }{\partial t} \frac{\partial f(\alpha ,\beta )}{\partial \alpha }}{g(\alpha )} - \frac{\frac{\partial \alpha }{\partial t}h(\alpha , \beta ) \frac{dg(\alpha )}{d\alpha }}{g(\alpha )^2} \right) } \\ &\quad + \frac{- h(\alpha , \beta )\frac{dg(\alpha )}{d\alpha }g(\alpha )^{-2}\frac{\partial ^2 \alpha }{\partial a \partial t}}{\left( \frac{\frac{\partial \alpha }{\partial t} \frac{\partial f(\alpha ,\beta )}{\partial \alpha }}{g(\alpha )} - \frac{\frac{\partial \alpha }{\partial t}h(\alpha , \beta ) \frac{dg(\alpha )}{d\alpha }}{g(\alpha )^2} \right) } \end{aligned}$$

$$\begin{aligned} &= \frac{ \frac{\partial ^2 \alpha }{\partial a\partial t} \frac{\partial f(\alpha ,\beta )}{\partial \alpha } g(\alpha )^{-1} + \frac{\partial \alpha }{\partial t} \left( \frac{\partial ^2 f(\alpha ,\beta )}{\partial \alpha ^2} g(\alpha )^{-1} - \frac{\partial f(\alpha ,\beta )}{\partial \alpha }g(\alpha )^{-2} \frac{dg}{d\alpha } \frac{\partial \alpha }{\partial a} \right) }{\left( \frac{\frac{\partial \alpha }{\partial t} \frac{\partial f(\alpha ,\beta )}{\partial \alpha }}{g(\alpha )} - \frac{\frac{\partial \alpha }{\partial t}h(\alpha , \beta ) \frac{dg(\alpha )}{d\alpha }}{g(\alpha )^2} \right) } \\ &\quad + \frac{ -\frac{\partial \alpha }{\partial t} \left[ h(\alpha , \beta ) \left( \frac{\partial \alpha }{\partial a} \frac{d^2 g(\alpha )}{d\alpha ^2} g(\alpha )^{-2} - 2 \left( \frac{dg(\alpha )}{d\alpha } \right) ^2 g(\alpha )^{-3} \frac{\partial \alpha }{\partial a} \right) + \frac{\partial h(\alpha , \beta )}{\partial \alpha } \frac{\partial \alpha }{\partial a} \frac{dg(\alpha )}{d\alpha } g(\alpha )^{-2} \right] }{\left( \frac{\frac{\partial \alpha }{\partial t} \frac{\partial f(\alpha ,\beta )}{\partial \alpha }}{g(\alpha )} - \frac{\frac{\partial \alpha }{\partial t}h(\alpha , \beta ) \frac{dg(\alpha )}{d\alpha }}{g(\alpha )^2} \right) } \\ &\quad + \frac{- h(\alpha , \beta )\frac{dg(\alpha )}{d\alpha }g(\alpha )^{-2}\frac{\partial ^2 \alpha }{\partial a \partial t}}{\left( \frac{\frac{\partial \alpha }{\partial t} \frac{\partial f(\alpha ,\beta )}{\partial \alpha }}{g(\alpha )} - \frac{\frac{\partial \alpha }{\partial t}h(\alpha , \beta ) \frac{dg(\alpha )}{d\alpha }}{g(\alpha )^2} \right) }\\&= \frac{ \frac{\partial ^2 \alpha }{\partial a\partial t} \frac{\partial f(\alpha ,\beta )}{\partial \alpha } g(\alpha )^{-1} + \frac{\partial \alpha }{\partial t} \left( \frac{\partial ^2 f(\alpha ,\beta )}{\partial \alpha ^2} g(\alpha )^{-1} - \frac{\partial f(\alpha ,\beta )}{\partial \alpha }g(\alpha )^{-2} \frac{dg}{d\alpha } \frac{\partial \alpha }{\partial a} \right) }{\left( \frac{\frac{\partial \alpha }{\partial t} \frac{\partial h(\alpha , \beta )}{\partial \alpha }g(\alpha ) - \frac{\partial \alpha }{\partial t} h(\alpha , \beta ) \frac{dg(\alpha )}{d\alpha }}{g(\alpha )^2} \right) } \\ &\quad + \frac{ -\frac{\partial \alpha }{\partial t} \left[ h(\alpha , \beta ) \left( \frac{\partial \alpha }{\partial a} \frac{d^2 g(\alpha )}{d\alpha ^2} g(\alpha )^{-2} - 2 \left( \frac{dg(\alpha )}{d\alpha } \right) ^2 g(\alpha )^{-3} \frac{\partial \alpha }{\partial a} \right) + \frac{\partial h(\alpha , \beta )}{\partial \alpha } \frac{\partial \alpha }{\partial a} \frac{dg(\alpha )}{d\alpha } g(\alpha )^{-2} \right] }{\left( \frac{\frac{\partial \alpha }{\partial t} \frac{\partial h(\alpha , \beta )}{\partial \alpha }g(\alpha ) - \frac{\partial \alpha }{\partial t} h(\alpha , \beta ) \frac{dg(\alpha )}{d\alpha }}{g(\alpha )^2} \right) } \\ &\quad + \frac{- h(\alpha , \beta )\frac{dg(\alpha )}{d\alpha }g(\alpha )^{-2}\frac{\partial ^2 \alpha }{\partial a \partial t}}{\left( \frac{\frac{\partial \alpha }{\partial t} \frac{\partial h(\alpha , \beta )}{\partial \alpha }g(\alpha ) - \frac{\partial \alpha }{\partial t} h(\alpha , \beta ) \frac{dg(\alpha )}{d\alpha }}{g(\alpha )^2} \right) } \\ &= \frac{ \frac{\partial ^2 \alpha }{\partial a\partial t} \frac{\partial f(\alpha ,\beta )}{\partial \alpha } g(\alpha ) + \left( \frac{\partial \alpha }{\partial t}\frac{\partial ^2 f(\alpha ,\beta )}{\partial \alpha ^2} g(\alpha ) - \frac{\partial \alpha }{\partial t}\frac{\partial f(\alpha ,\beta )}{\partial \alpha } \frac{dg}{d\alpha } \frac{\partial \alpha }{\partial a} \right) }{\frac{\partial \alpha }{\partial t} \frac{\partial h(\alpha , \beta )}{\partial \alpha }g(\alpha ) - \frac{\partial \alpha }{\partial t} h(\alpha , \beta ) \frac{dg(\alpha )}{d\alpha }} \\ &\quad + \frac{ -\frac{\partial \alpha }{\partial t} \left[ h(\alpha , \beta ) \left( \frac{\partial \alpha }{\partial a} \frac{d^2 g(\alpha )}{d\alpha ^2} - 2 \left( \frac{dg(\alpha )}{d\alpha } \right) ^2 g(\alpha )^{-1} 
\frac{\partial \alpha }{\partial a} \right) + \frac{\partial h(\alpha , \beta )}{\partial \alpha } \frac{\partial \alpha }{\partial a} \frac{dg(\alpha )}{d\alpha } \right] }{\frac{\partial \alpha }{\partial t} \frac{\partial h(\alpha , \beta )}{\partial \alpha }g(\alpha ) - \frac{\partial \alpha }{\partial t} h(\alpha , \beta ) \frac{dg(\alpha )}{d\alpha }} \\ &\quad + \frac{- h(\alpha , \beta )\frac{dg(\alpha )}{d\alpha }\frac{\partial ^2 \alpha }{\partial a \partial t}}{\frac{\partial \alpha }{\partial t} \frac{\partial h(\alpha , \beta )}{\partial \alpha }g(\alpha ) - \frac{\partial \alpha }{\partial t} h(\alpha , \beta ) \frac{dg(\alpha )}{d\alpha }} \\ &= \frac{ \frac{\partial ^2 \alpha }{\partial a\partial t} \frac{\partial f(\alpha ,\beta )}{\partial \alpha } g^2(\alpha ) + \frac{\partial \alpha }{\partial t}\frac{\partial ^2 f(\alpha ,\beta )}{\partial \alpha ^2} g(\alpha )^2 - \frac{\partial \alpha }{\partial t}\frac{\partial f(\alpha ,\beta )}{\partial \alpha } \frac{dg(\alpha )}{d\alpha } \frac{\partial \alpha }{\partial a}g(\alpha )}{g(\alpha )\frac{\partial \alpha }{\partial t}\left( \frac{\partial h(\alpha , \beta )}{\partial \alpha }g(\alpha ) - h(\alpha , \beta ) \frac{dg(\alpha )}{d\alpha }\right) } \\ &\quad + \frac{ - \left[ h(\alpha , \beta ) \left( \frac{\partial \alpha }{\partial t}\frac{\partial \alpha }{\partial a} \frac{d^2 g(\alpha )}{d\alpha ^2}g(\alpha ) - 2 \frac{\partial \alpha }{\partial t}\left( \frac{dg(\alpha )}{d\alpha } \right) ^2 \frac{\partial \alpha }{\partial a} \right) + \frac{\partial \alpha }{\partial t} \frac{\partial h(\alpha , \beta )}{\partial \alpha } \frac{\partial \alpha }{\partial a} \frac{dg(\alpha )}{d\alpha }g(\alpha ) \right] }{g(\alpha )\frac{\partial \alpha }{\partial t}\left( \frac{\partial h(\alpha , \beta )}{\partial \alpha }g(\alpha ) - h(\alpha , \beta ) \frac{dg(\alpha )}{d\alpha }\right) } \\ &\quad + \frac{- h(\alpha , \beta )\frac{dg(\alpha )}{d\alpha }\frac{\partial ^2 \alpha }{\partial a \partial t}g(\alpha )}{g(\alpha )\frac{\partial \alpha }{\partial t}\left( \frac{\partial h(\alpha , \beta )}{\partial \alpha }g(\alpha ) - h(\alpha , \beta ) \frac{dg(\alpha )}{d\alpha }\right) } \\ &= \frac{h(\alpha , \beta ) \left[ - \frac{\partial \alpha }{\partial t}\frac{\partial \alpha }{\partial a}g(\alpha )\frac{d^2 g(\alpha )}{d \alpha ^2} + 2 \frac{\partial \alpha }{\partial t} \frac{\partial \alpha }{\partial a} \left( \frac{dg(\alpha )}{d \alpha } \right) ^2 - \frac{\partial ^2 \alpha }{\partial a \partial t}g(\alpha )\frac{dg(\alpha )}{d\alpha } \right] }{g(\alpha )\frac{\partial \alpha }{\partial t}\left( \frac{\partial h(\alpha , \beta )}{\partial \alpha }g(\alpha ) - h(\alpha , \beta ) \frac{dg(\alpha )}{d\alpha }\right) } \\ &\quad + \frac{g(\alpha ) \left[ g(\alpha ) \frac{\partial ^2 \alpha }{\partial a \partial t} \frac{\partial h(\alpha , \beta )}{\partial \alpha } + \frac{\partial \alpha }{\partial t} \frac{\partial ^2 h(\alpha , \beta )}{\partial a^2}g(\alpha ) - \frac{\partial \alpha }{\partial a}\frac{\partial \alpha }{\partial t} \frac{dg(\alpha )}{d\alpha }\frac{\partial h(\alpha , \beta )}{\partial \alpha } - \frac{\partial \alpha }{\partial t}\frac{\partial \alpha }{\partial a} \frac{\partial h(\alpha , \beta )}{\partial \alpha }\frac{dg(\alpha )}{d(\alpha )} \right] }{g(\alpha )\frac{\partial \alpha }{\partial t}\left( \frac{\partial h(\alpha , \beta )}{\partial \alpha }g(\alpha ) - h(\alpha , \beta ) \frac{dg(\alpha )}{d\alpha }\right) } \\ &= \frac{g(\alpha ) \biggl [ g(\alpha ) \left( \frac{\partial ^2 \alpha }{\partial a \partial t} \frac{\partial h(\alpha , \beta )}{\partial \alpha } + \frac{\partial \alpha }{\partial t} \frac{\partial \alpha }{\partial a} \frac{\partial ^2 h(\alpha , \beta )}{\partial \alpha ^2}\right) - 2 \frac{\partial \alpha }{\partial t} \frac{\partial \alpha }{\partial a} \frac{\partial h(\alpha , \beta )}{\partial \alpha } \frac{d g(\alpha )}{d \alpha } \biggr ]}{ \frac{\partial \alpha }{\partial t} \ g(\alpha ) \bigr ) \bigl ( g(\alpha ) \frac{\partial h(\alpha , \beta )}{\partial \alpha } - h(\alpha , \beta ) \frac{d g(\alpha )}{d \alpha }} \\ &\quad + \frac{h(\alpha , \beta ) \left( -\frac{\partial \alpha }{\partial t} \frac{\partial \alpha }{\partial a} g(\alpha ) \frac{d^2 g}{d \alpha ^2} + 2 \frac{\partial \alpha }{\partial t} \frac{\partial \alpha }{\partial a} \bigl ( \frac{d g(\alpha )}{d \alpha } \bigr )^2 - \frac{\partial ^2 \alpha }{\partial a \partial t} g(\alpha ) \frac{d g(\alpha )}{d \alpha } \right) }{ \frac{\partial \alpha }{\partial t} \ g(\alpha ) \bigr ) \bigl ( g(\alpha ) \frac{\partial h(\alpha , \beta )}{\partial \alpha } - h(\alpha , \beta ) \frac{d g(\alpha )}{d \alpha } } \end{aligned}$$

$$\square$$

### Appendix 1.5: Proof of Eq. (32)

$$\begin{aligned} \begin{aligned} u_b = \frac{\frac{\partial h(\alpha , \beta )}{\partial \beta } \frac{d g(\alpha )}{d \alpha } - g(\alpha ) \frac{\partial ^2 h(\alpha , \beta )}{\partial \alpha \partial \beta } }{h(\alpha , \beta )\frac{d g(\alpha )}{d \alpha } - g(\alpha ) \frac{\partial h(\alpha , \beta )}{\partial \alpha }}, \end{aligned} \end{aligned}$$

#### Proof

$$\begin{aligned} u_\beta&= \frac{\partial }{\partial \beta } \ln f_T(t; a, \beta ) = \frac{\partial }{\partial \beta } \left( \ln \left( \frac{1}{g(\alpha )^2} \biggl [ \frac{\partial \alpha }{\partial t} \biggl ( g(\alpha ) \frac{\partial h(\alpha , \beta )}{\partial \alpha } - h(\alpha , \beta ) \frac{d g(\alpha )}{d \alpha } \biggr ) \biggr ] \right) \right) \\ &= \frac{\partial }{\partial \beta } \left( \ln \left( \frac{\frac{\partial \alpha }{\partial t} \frac{\partial f(\alpha ,\beta )}{\partial \alpha }}{g(\alpha )} - \frac{\frac{\partial \alpha }{\partial t}h(\alpha , \beta ) \frac{dg(\alpha )}{d(\alpha )}}{g(\alpha )^2} \right) \right) \\ &= \frac{\frac{\partial }{\partial \beta }\left( \frac{\partial \alpha }{\partial t} \frac{\partial f(\alpha ,\beta )}{\partial \alpha } g(\alpha )^{-1} - \frac{\partial \alpha }{\partial t} h(\alpha , \beta ) \frac{dg(\alpha )}{d\alpha }g(\alpha )^{-2} \right) }{\left( \frac{\frac{\partial \alpha }{\partial t} \frac{\partial f(\alpha ,\beta )}{\partial \alpha }}{g(\alpha )} - \frac{\frac{\partial \alpha }{\partial t}h(\alpha , \beta ) \frac{dg(\alpha )}{d\alpha }}{g(\alpha )^2} \right) } \\ &= \frac{\left( \frac{\partial \alpha }{\partial t} \frac{\partial ^2 f(\alpha ,\beta )}{\partial \alpha \partial \beta } g(\alpha )^{-1} - \frac{\partial \alpha }{\partial t} \frac{\partial h(\alpha , \beta )}{\partial \beta } \frac{dg(\alpha )}{d\alpha }g(\alpha )^{-2} \right) }{\left( \frac{\frac{\partial \alpha }{\partial t} \frac{\partial f(\alpha ,\beta )}{\partial \alpha }}{g(\alpha )} - \frac{\frac{\partial \alpha }{\partial t}h(\alpha , \beta ) \frac{dg(\alpha )}{d\alpha }}{g(\alpha )^2} \right) } \\ &= \frac{\left( \frac{\partial \alpha }{\partial t} \frac{\partial ^2 f(\alpha ,\beta )}{\partial \alpha \partial \beta } g(\alpha )^{-1} - \frac{\partial \alpha }{\partial t} \frac{\partial h(\alpha , \beta )}{\partial \beta } \frac{dg(\alpha )}{d\alpha }g(\alpha )^{-2} \right) }{\left( \frac{\frac{\partial \alpha }{\partial t} \frac{\partial h(\alpha , \beta )}{\partial \alpha }g(\alpha ) - \frac{\partial \alpha }{\partial t} h(\alpha , \beta ) \frac{dg(\alpha )}{d\alpha }}{g(\alpha )^2} \right) } \\ &= \frac{\frac{\partial \alpha }{\partial t} \frac{\partial ^2 f(\alpha ,\beta )}{\partial \alpha \partial \beta } g(\alpha ) - \frac{\partial \alpha }{\partial t} \frac{\partial h(\alpha , \beta )}{\partial \beta } \frac{dg(\alpha )}{d\alpha }}{\frac{\partial \alpha }{\partial t} \frac{\partial h(\alpha , \beta )}{\partial \alpha }g(\alpha ) - \frac{\partial \alpha }{\partial t} h(\alpha , \beta ) \frac{dg(\alpha )}{d\alpha }} \\ &= \frac{\frac{\partial ^2 f(\alpha ,\beta )}{\partial \alpha \partial \beta } g(\alpha ) - \frac{\partial h(\alpha , \beta )}{\partial \beta } \frac{dg(\alpha )}{d\alpha }}{\frac{\partial h(\alpha , \beta )}{\partial \alpha }g(\alpha ) - h(\alpha , \beta ) \frac{dg(\alpha )}{d\alpha }} \\ &= \frac{\frac{\partial h(\alpha , \beta )}{\partial \beta } \frac{d g(\alpha )}{d \alpha } - g(\alpha ) \frac{\partial ^2 h(\alpha , \beta )}{\partial \alpha \partial \beta } }{h(\alpha , \beta )\frac{d g(\alpha )}{d \alpha } - g(\alpha ) \frac{\partial h(\alpha , \beta )}{\partial \alpha }} \end{aligned}$$

$$\square$$

### Appendix 1.6: Proof of Eq. (34)

$$\begin{aligned} u_\alpha = - \frac{\frac{\partial \alpha }{\partial a} (h(\alpha , \beta ) \frac{d g(\alpha )}{d \alpha } - g(\alpha )\frac{\partial h(\alpha , \beta )}{\partial \alpha } )}{g(\alpha )(h(\alpha , \beta ) - g(\alpha ))} \end{aligned}$$

#### Proof

$$\begin{aligned} u_a&= \frac{\partial }{\partial a} \ln S_T(t; a, \beta ) = \frac{\partial }{\partial a} \ln \left( 1 - \frac{h(\alpha , \beta )}{g(\alpha )} \right) = \frac{\frac{\partial }{\partial a}\left( 1 - \frac{h(\alpha , \beta )}{g(\alpha )}\right) }{1 - \frac{h(\alpha , \beta )}{g(\alpha )}} \\ &=\quad \quad \frac{- \left[ \frac{\partial h(\alpha , \beta )}{\partial \alpha } \frac{\partial \alpha }{\partial a}g(\alpha )^{-1} + h(\alpha , \beta ) \frac{\partial }{\partial a}\left( g(\alpha )^{-1}\right) \right] }{1 - \frac{h(\alpha , \beta )}{g(\alpha )}} \\ &=\quad \quad \frac{- \frac{\partial h(\alpha , \beta )}{\partial \alpha } \frac{\partial \alpha }{\partial a}g(\alpha )^{-1} + h(\alpha , \beta ) g(\alpha )^{-2}\frac{dg(\alpha )}{d \alpha }\frac{\partial \alpha }{\partial a}}{1 - \frac{h(\alpha , \beta )}{g(\alpha )}} \\&=\quad \quad \frac{- \frac{\partial h(\alpha , \beta )}{\partial \alpha } \frac{\partial \alpha }{\partial a}g(\alpha )^{-1} + h(\alpha , \beta ) g(\alpha )^{-2}\frac{dg(\alpha )}{d \alpha }\frac{\partial \alpha }{\partial a}}{\frac{g(\alpha ) - h(\alpha , \beta )}{g(\alpha )}} \\ &=\quad \quad \frac{- \frac{\partial h(\alpha , \beta )}{\partial \alpha } \frac{\partial \alpha }{\partial a} + h(\alpha , \beta ) g(\alpha )^{-1}\frac{dg(\alpha )}{d \alpha }\frac{\partial \alpha }{\partial a}}{g(\alpha ) - h(\alpha , \beta )}\\ &= \quad \quad = - \frac{\frac{\partial \alpha }{\partial a} (h(\alpha , \beta ) \frac{d g(\alpha )}{d \alpha } - g(\alpha )\frac{\partial h(\alpha , \beta )}{\partial \alpha } )}{g(\alpha )(h(\alpha , \beta ) - g(\alpha ))} \end{aligned}$$

$$\square$$

### Appendix 1.7: Approximation for the gamma cdf

The approximation $P(t; at, \beta ) \approx I(at, \beta )$ is obtained in the R package VGAM (Yee 2010) as a series expansion for $at \le \beta \le 1$ and for $\beta < at$:

$$\begin{aligned} I(\beta , at) = fG \quad \quad \text {with} \quad f = \frac{\beta ^{at}}{\varGamma (at+1)e^{\beta }} \quad \text {and} \quad G = \sum _{n=0}^\infty C_n \end{aligned}$$

where $C_n = \frac{\beta }{at+n}C_{n-1}$ for $n=1,2,\ldots$ and $C_0(\beta , at)=1$; otherwise, the approximation is made with a continued fraction expansion,

$$\begin{aligned} I(\beta , at) =&\ 1 - fG \quad \quad \text {with} \quad f = \frac{\beta ^{at}}{\varGamma (at)e^{\beta }} \end{aligned}$$

$$\begin{aligned} \text {and} \quad&\ G = \frac{1}{\beta } \left( 1 + \frac{at-1}{(2-at+\beta )+} \frac{at-2}{(4-at+\beta )+} \frac{2(at-3)}{(6-at+\beta )+} \dots \right) \end{aligned}$$

where the plus signs in the denominators of the continued fraction indicate that each term is added to the subsequent fraction in the sequence. This is a standard notation for continued fractions:

$$\begin{aligned} a_0 + \frac{b_1}{a_1+}\frac{b_2}{a_2+}\frac{b_3}{a_3+} = a_0 + \frac{b_1}{a_1 + \frac{b_2}{a_2 + \frac{b_3}{a_3 + \cdots }}} \end{aligned}$$

Specifically, the *n*th convergent $G_n = \frac{A_n}{B_n} = \frac{t}{\beta } \biggl ( 1 + \frac{a_1}{b_1+} \frac{a_2}{b_2+}\ldots \frac{a_n}{b_n} \biggr )$ is obtained recursively using

$$\begin{aligned} \begin{aligned} A_n = b_n A_{n-1} + a_n A_{n-2} \\ B_n = b_n B_{n-1} + a_n B_{n-2} \end{aligned} \end{aligned}$$

with

$$\begin{aligned} \begin{aligned}&a_n = (n-1)(at-n) \\&b_n = 2n-at+\beta \\&A_0=1, \quad B_0=\beta , \quad A_1=\beta +1, \quad B_1=\beta (2-at+\beta ). \end{aligned} \end{aligned}$$

The derivatives with respect to $\alpha = at$ are obtained using the chain rule in *f* and *G*:

$$\begin{aligned} \begin{aligned}&\frac{\partial }{\partial \alpha }I(\alpha , \beta ) = G\frac{\partial f}{\partial \alpha } + f\frac{\partial G}{\partial \alpha } \quad \text {for the series expansion} \bigl ( \alpha \le \beta \le 1, \, \beta < \alpha \bigr ) \\&\frac{\partial }{\partial \alpha }I(\alpha , \beta ) = - f\frac{\partial G}{\partial \alpha } - G\frac{\partial f}{\partial \alpha } \quad \text {for the continued fraction expansion}. \end{aligned} \end{aligned}$$

These expressions have been adapted from Moore (2018), to which we refer for further details.

### Appendix 1.8: Data generation for the simulated scenarios

Given that the distribution from which we need to sample is non-standard, we adopted a rejection sampling algorithm to generate *n* data-points. The process begins by sampling a candidate *z* from a proposal distribution *q*(*z*), sampling from which is easier compared to the target distribution *p*(*z*), and such that it satisfies $p(z) \le M\cdot q(z)$ for some constant $M \ge 1$. The acceptance probability for the candidate is $\rho (z) = \frac{p(z)}{M \cdot q(z)}$. A uniform random number is drawn from a standard uniform *U*(0, 1), and the candidate *z* is accepted if $u \le \rho (z)$; otherwise, it is rejected. This is repeated until the set of accepted candidates contains *n* items; of course, the efficiency of the algorithm is highly dependant on the choice of *q*(*z*) and *M*. For more details on sampling through rejection sampling, we refer to Robert and Casella (2004).

In our case, the target distribution is different for each individual, as it depends on parameters which vary with the covariates. Given the challenge of finding a single proposal distribution that works well across all data points, we opted for a uniform distribution, which is dynamically determined based on the characteristics of each individual’s target distribution to ensure it properly bounds the target distribution from above. To do so, we begin by determining the range of the proposal distribution. For each data point we calculate the upper limit $z^\star$ of the range of the uniform distribution using the highest density interval method: $z^\star$ is chosen such that it includes approximately 99.5% of the probability mass of the target distribution. Next, we compute the maximum value of the target density, $\hbox {max}_p(z)$, within the range $(0,z^\star ]$, which is crucial to scale the proposal distribution. The proposal distribution is then uniform over the interval $[0.001, z^\star ]$, i.e. with a pdf equal to $\frac{1}{z^\star - 0.001}$ within the interval and 0 elsewhere. We calculate a scaling factor $c = \bigl ( {\hbox {max}}_{p}(z)\bigr /{\frac{1}{z^\star - 0.001}} \bigr ) \times 1.3$ to ensure that this proposal appropriately bounds the target, where the multiplication by 1.3 allows for extra variability. The final proposal density is therefore

$$\begin{aligned} q(z) = \frac{1}{z^\star - 0.001} \times c \quad \quad \hbox {with} \quad c = \biggl ( {\hbox {max}}_{p}(z)\biggr /{\frac{1}{z^\star - 0.001}} \biggr ) \times 1.3 \end{aligned}$$

The algorithm then proceeds with rejection sampling. To compute the constant *M*, it evaluates the ratio of the target distribution to the proposal distribution across a range of values within the proposal’s interval, and sets $M = \hbox {max} \bigl ( \frac{p(z)}{q(z)} \bigr )$ so that the condition $p(z) \le M\cdot q(z)$ is always met. During the sampling process, a candidate sample $z_{\hbox {cand}}$ is drawn from the proposal distribution, and it is accepted if a uniformly drawn random number *u* satisfies $u \le \rho (z_{\hbox {cand}})$.

### Appendix 1.9: Covariates for the lithium-ion dataset

See Table 4 for definitions and descriptions of the covariates for the lithium-ion dataset, which have been extracted from the row sensor data of the batteries.

**Table 4:** Battery-related covariates included in the analysis of the Toyota Research Institute dataset

Variable name	Formula	Description
start.cap	$C_0$	First observed capacity value at beginning of operations
avg.avg.voltage	$\frac{1}{n_{\tiny {\text {cycles}}}}\sum _i^{n_{\tiny {\text {cycles}}}} \bar{V}_i$	Average of the average voltage of each discharge cycle
avg.var.voltage	$\frac{1}{n_{\tiny {\text {cycles}}}}\sum _i^{n_{\tiny {\text {cycles}}}} \text {var}(V)_i$	Average of the voltage variance of each discharge cycle
avg.delta.voltage	$\frac{1}{n_{\tiny {\text {cycles}}}}\sum _i^{n_{\tiny {\text {cycles}}}} \varDelta V_i$	Average of the delta voltage of each discharge cycle
avg.avg.current	$\frac{1}{n_{\tiny {\text {cycles}}}}\sum _i^{n_{\tiny {\text {cycles}}}} \bar{c}_i$	Average of the average current of each discharge cycle
avg.var.current	$\frac{1}{n_{\tiny {\text {cycles}}}}\sum _i^{n_{\tiny {\text {cycles}}}} \text {var}(c)_i$	Average of the current variance of each discharge cycle
max.var.current	$\text {max} (\text {var}(c)_i)$	Max current variance over the discharge cycles
delta_charge_1	$\bar{c}_{\tiny {\text {before-first}}} - \bar{c}_{\tiny {\text {after-first}}}$	Difference between mean current before and after the 1st current change in a charge cycle
delta_charge_2	$\bar{c}_{\tiny {\text {before-second}}} - \bar{c}_{\tiny {\text {after-second}}}$	Difference between mean current before and after the 2nd current change in a charge cycle
delta_temp	$\bar{T}^{\tiny {\text {max}}}_{(n-19):n} - \bar{T}^{\tiny {\text {max}}}_{1:20}$	Difference between mean max temp in the first and last 20 cycles
temp.extremes	$\sum _j^{N_{\tiny {\text {values}}}} I(T_j^{\tiny {\text {max}}}> \bar{T}^{\tiny {\text {max}}} + 2$ $\quad \quad \quad \quad | T_j^{\tiny {\text {min}}} < \bar{T}^{\tiny {\text {min}}} - 2)$	How many times $T^{\tiny {\text {max}}}> \bar{T}^{\tiny {\text {max}}} + 2$ or $T^{\tiny {\text {min}}} < \bar{T}^{\tiny {\text {min}}} - 2$

## Footnotes

1 Hardware/software configuration: Intel ® Core™ i7-8550U CPU; Memory 16 G, Programming Language R 4.1.2.
