# Supplementary material for: Lifetime analysis with monotonic degradation: a boosted first hitting time model based on a homogeneous gamma process
Source: Lifetime Data Anal. 2025 Apr 5;31(2):300–39. doi: 10.1007/s10985-025-09648-z (PMC12043765; doi:10.1007/s10985-025-09648-z)
Supplement: Supplementary file 1 — (pdf 384 KB) [file 10985_2025_9648_MOESM1_ESM.pdf]

Supplementary Material for

Lifetime analysis with monotonic degradation: a boosted First Hitting Time model based on a homogeneous gamma process

Clara Bertinelli Salucci[*†] Azzeddine Bakdi[‡] Ingrid Kristine Glad [*]
Bo Henry Lindqvist [§] Erik Vanem [¶*] Riccardo De Bin [*]

Contents

1. Simulation Study

2. Predictive performance of the models on the simulated data

[*]Corresponding author
[†]University of Oslo, Department of Mathematics
[‡]Corvus Energy
[§]Norwegian University of Science and Technology, Department of Mathematical Sciences
[¶]DNV Group Technology and Research

Simulation Study

We have generated 1000 samples of sizes $n_1 = 100$ and $n_2 = 500$ with about 10%, 50% and 90% of censored data[1] for each of the two examples presented in the article. The estimated parameters are shown in Figure 1 for the first and Figures 2, 3, and 4 for Example 2, demonstrating how sample size and censoring percentage impact the accuracy and variability of parameter estimates.

In Figure 1, as the sample size increases from 100 to 500 the estimates become more accurate and consistent, with the boxplots narrowing and centering closely around the true values, represented by red points. This suggests that larger sample sizes provide more reliable estimates, as expected. On the other hand, increasing the censoring percentage from 10% to 90% leads to greater variability and bias in the estimates, particularly in smaller samples, where the boxplots are wider and we can observe more deviation from the true values. However, even in the most extreme scenario, with only 100 data points and 90% censoring, the estimates remain within a reasonable range and the median estimates are mostly close to the true values, indicating that the estimation process is still robust enough to provide useful information despite the challenging conditions. This robustness suggests that while larger sample sizes and lower censoring percentages are preferable, the methodology employed here can still yield reasonable estimates under less-than-ideal circumstances.

Comparing Figures 2, 3 and 4 allows us to observe the effect of increasing censoring proportions in Example 2. These plots illustrate that, as censoring increases from 10% to 50% and 90%, some important trends emerge. The most noticeable effect is the widening of the distribution of parameter estimates, particularly for the covariate coefficients. This suggests that higher levels of censoring reduce the precision of the estimates due to the loss of information when more data points are censored, which is also confirmed by the increased presence of outliers for higher censoring rates. Additionally, the red points show an increasing deviation from the centre of the boxplots as censoring rises, particularly in the smaller sample size ($n = 100$, left panel). This shift indicates that higher censoring rates are likely to introduce bias into the parameter estimates. Within each figure, comparing the results for different sample sizes reveals the impact of increasing the sample size from 100 to 500 data points. With a larger sample size, the variability of the parameter estimates decreases, as indicated by the narrower boxplots. This suggests that a larger sample size provides more stable and precise estimates, reducing the impact of sampling variability. Furthermore, in scenarios with larger samples, the alignment between the red points and the centers of the boxplots improves. This implies that larger samples help mitigate the bias introduced by censoring: even in extreme situations, such as with 90% censoring, having a sufficiently large sample size can make the estimation method quite robust, maintaining on average the quality and reliability of the parameter estimates.

When comparing these findings with the scenario of Example 1, it is manifest that the algorithm performs significantly better in the former case. The method yields more stable and accurate parameter estimates in Example 1, as shown by tighter boxplots and fewer outliers, even with high levels of censoring. In contrast, in the second, sparser scenario the

[1] Corresponding to a censoring exponential rate of, respectively, 0.1, 0.88 and 5.5 for Example 1, and of 3, 25 and 120 for Example 2

presence of many covariates with smaller effect or zero coefficient introduces more noise, leading to greater variability and less reliable estimates. The algorithm is clearly more robust when the underlying signal is strong; however, in Example 2, the algorithm struggles especially with higher censoring rates and smaller samples, implying that a larger sample size can counteract the increased variability and bias introduced by censoring and reduced signal.

Predictive performance of the models on the simulated data

Figures 5 and 6 illustrate the predictive performance of the models considered in the article (FHTgamma, FHTwiener, Cox, and the Kaplan Meier estimator) evaluated using integrated Brier scores and concordance indexes across 100 runs with data generated as from Example 1 in the article, with different levels of data censoring (10%, 50%, and 90%). As expected, the FHTgamma model consistently demonstrates superior predictive performance, given that it aligns with the data-generating mechanism. This superiority is evident across all levels of censoring. The Cox model also performs well, showing strong predictive capabilities that are only slightly inferior to those of the FHTgamma model. While the FHTwiener model does not perform as well as the FHTgamma model, it still significantly outperforms the Kaplan Meier estimator, indicating that even a model not perfectly aligned with the data generation process can provide substantial improvements over the non-parametric baseline model.

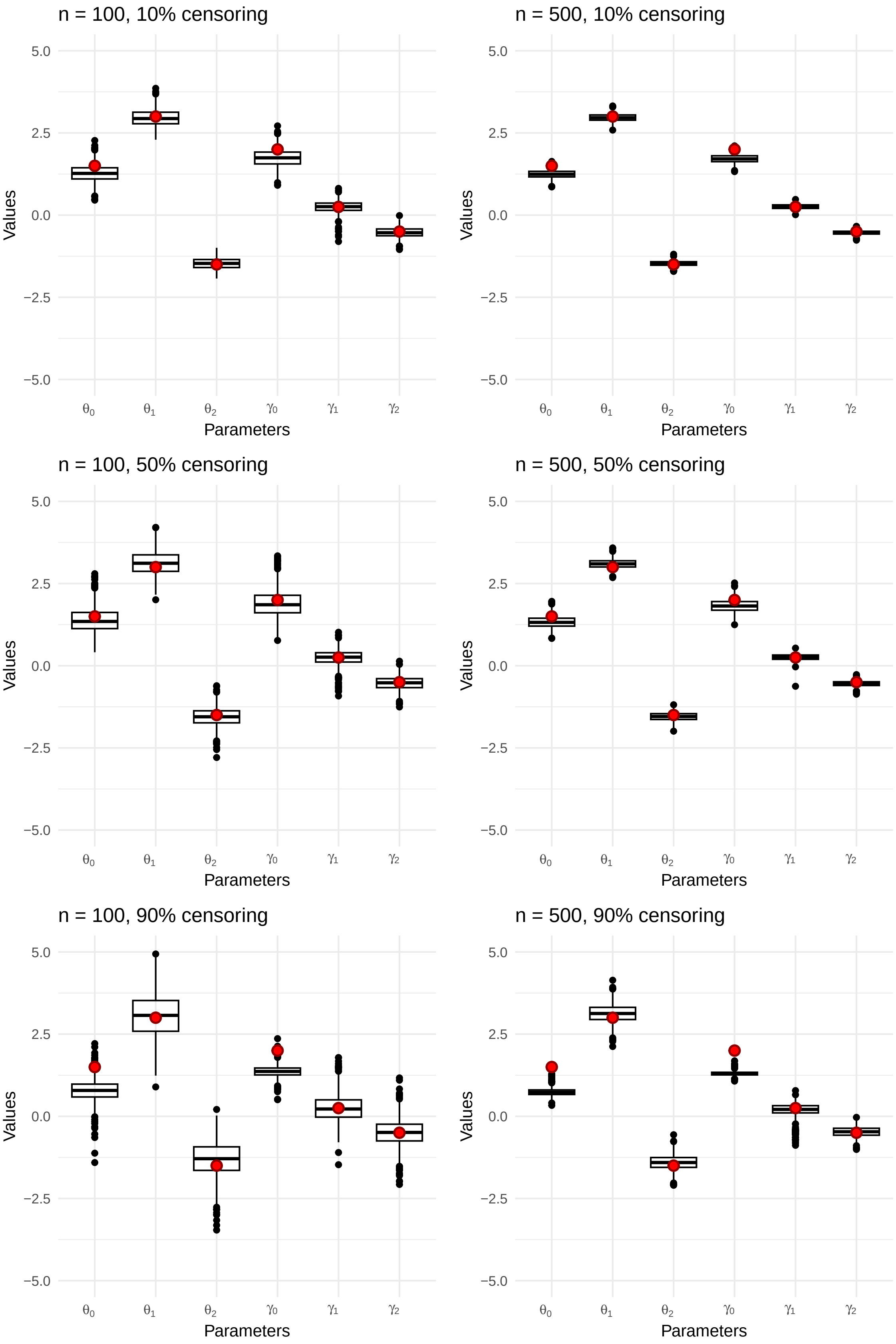

Figure 1: Effect of sample size ($n = 100$ and $n = 500$) and censoring percentage (10%, 50%, and 90%) on the distribution of parameter estimates across 1000 simulations of Example 1. The red points represent the true parameter values, while the boxplots show the variability of the estimates.

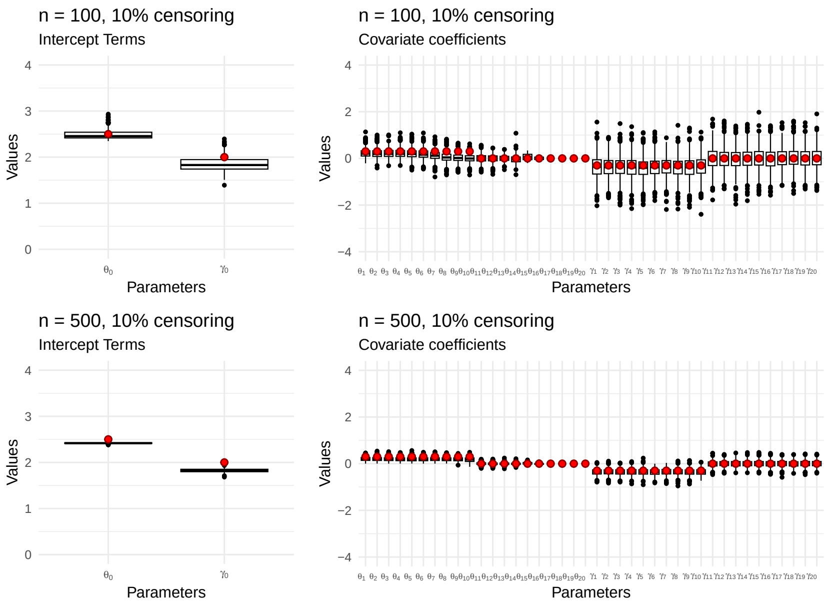

Figure 2: Effect of sample size ($n = 100$ and $n = 500$) on the distribution of parameter estimates across 1000 simulations of Example 2 with 10% censoring. The red points represent the true parameter values, while the boxplots show the variability of the estimates.

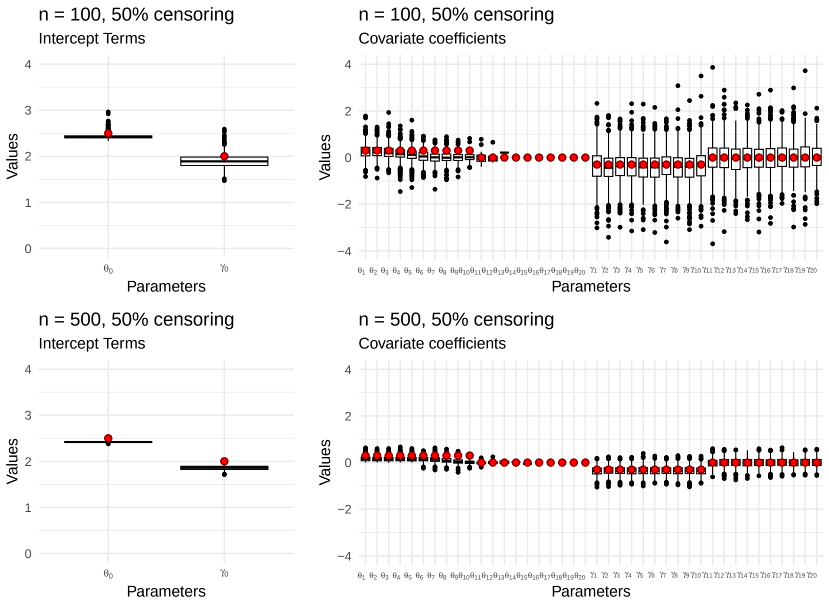

Figure 3: Effect of sample size ($n = 100$ and $n = 500$) on the distribution of parameter estimates across 1000 simulations of Example 2 with 50% censoring. The red points represent the true parameter values, while the boxplots show the variability of the estimates.

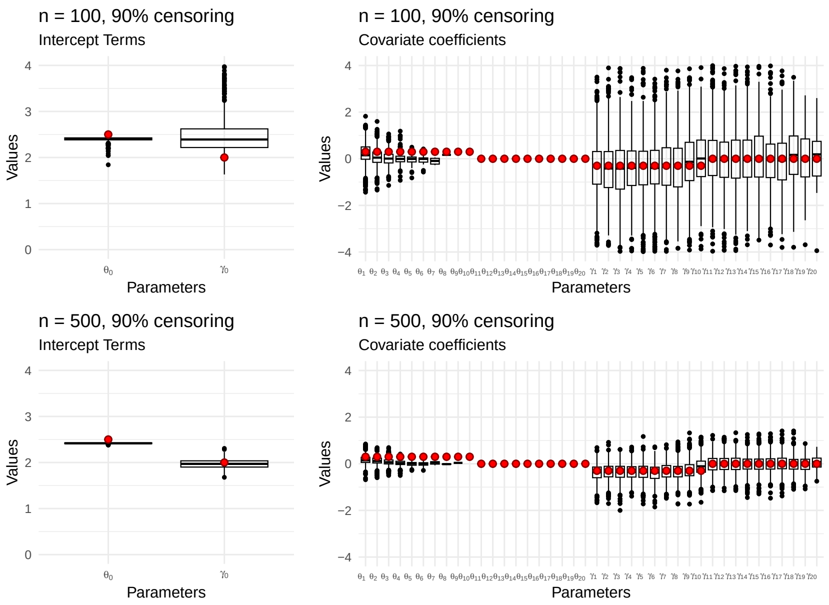

Figure 4: Effect of sample size ($n = 100$ and $n = 500$) on the distribution of parameter estimates across 1000 simulations of Example 2 with 90% censoring. The red points represent the true parameter values, while the boxplots show the variability of the estimates.

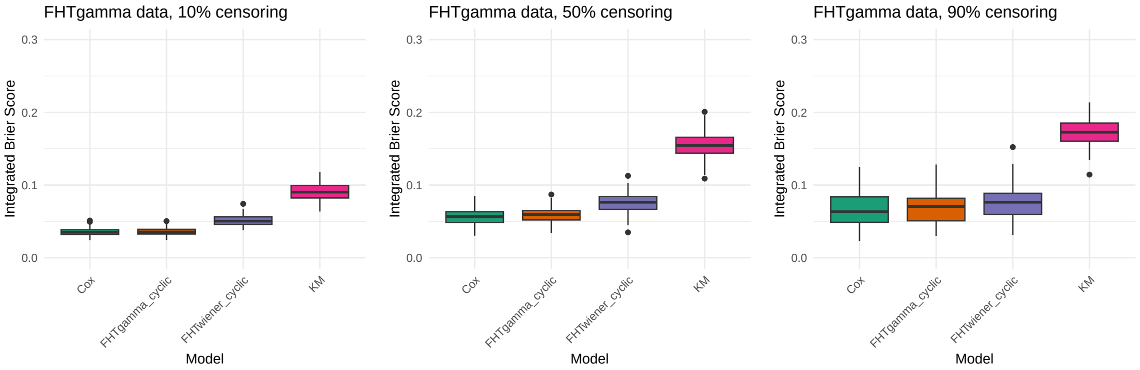

Figure 5: Integrated Brier scores for predictions from the different models (FHTgamma, FHTwiener, Cox, and the Kaplan Meier estimator as reference) for 100 runs. 500 observations are generated from a First Hitting Time density based on a homogeneous gamma process, with parameters as described in Example 1 in the article; two third are used for training and one third for testing.

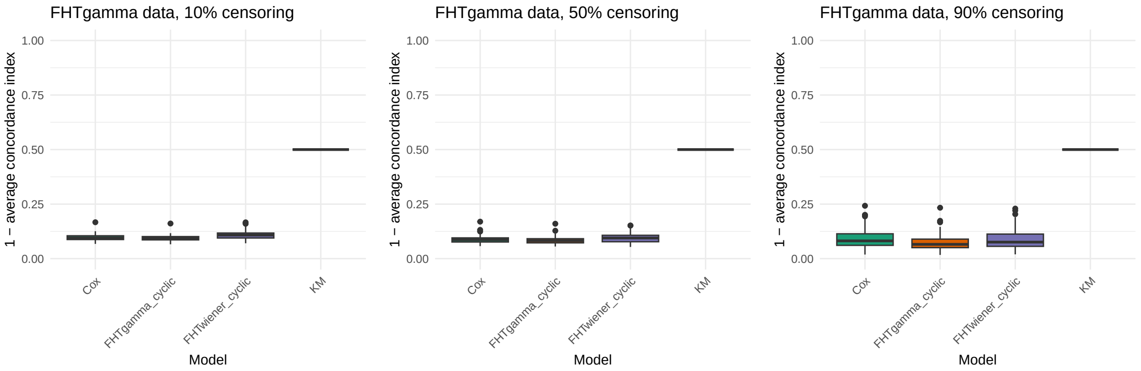

Figure 6: Average concordance indexes for predictions from the different models (FHTgamma, FHTwiener, Cox, and the Kaplan Meier estimator as reference) for 100 runs. 500 observations are generated from a First Hitting Time density based on a homogeneous gamma process, with parameters as described in Example 1 in the article; two third are used for training and one third for testing. Lower values indicate better performance.
